# Plant Terpenoids as Hit Compounds against Trypanosomiasis

**DOI:** 10.3390/ph15030340

**Published:** 2022-03-10

**Authors:** Raquel Durão, Cátia Ramalhete, Ana Margarida Madureira, Eduarda Mendes, Noélia Duarte

**Affiliations:** 1 Research Institute for Medicines (iMED.Ulisboa), Faculdade de Farmácia, Universidade de Lisboa, Av. Prof. Gama Pinto, 1649-003 Lisboa, Portugal; raquel-durao@campus.ul.pt (R.D.); catiar@uatlantica.pt (C.R.); afernand@ff.ulisboa.pt (A.M.M.); ermendes@ff.ulisboa.pt (E.M.); 2 ATLANTICA—Instituto Universitário, Fábrica da Pólvora de Barcarena, 2730-036 Barcarena, Portugal

**Keywords:** terpenoids, Trypanosoma, human African trypanosomiasis, sleeping disease, human American trypanosomiasis, Chagas disease

## Abstract

Human African trypanosomiasis (sleeping sickness) and American trypanosomiasis (Chagas disease) are vector-borne neglected tropical diseases, caused by the protozoan parasites *Trypanosoma brucei* and *Trypanosoma cruzi*, respectively. These diseases were circumscribed to South American and African countries in the past. However, human migration, military interventions, and climate changes have had an important effect on their worldwide propagation, particularly Chagas disease. Currently, the treatment of trypanosomiasis is not ideal, becoming a challenge in poor populations with limited resources. Exploring natural products from higher plants remains a valuable approach to find new hits and enlarge the pipeline of new drugs against protozoal human infections. This review covers the recent studies (2016–2021) on plant terpenoids, and their semi-synthetic derivatives, which have shown promising in vitro and in vivo activities against Trypanosoma parasites.

## 1. Introduction

Human African trypanosomiasis (sleeping sickness) and American trypanosomiasis (Chagas disease) are among the twenty Neglected Tropical Diseases (NTDs) defined as such by the World Health Organization (WHO). NTDs are a heterogeneous group of diseases including, among others, several parasitic, viral, and bacterial infections, responsible for high morbidity and mortality, and affecting more than one billion people globally [1,2,3]. Sometimes, the impact of NTDs on health can be underestimated because many infections are asymptomatic and associated with long incubation periods. Nevertheless, NTDs are recognized as a public health problem, particularly for people living in rural and conflict areas in developing countries [1]. These diseases are considered neglected due to the general lack of attention in developed countries and almost non-existent financial investment in the research and development of new drugs and vaccines [4]. In addition to all these problems, the COVID-19 pandemic has been affecting the programs of mass drug administration and other NTD control measures [5,6]. Currently, the pharmacological therapy of NTDs is not ideal, as it has some limitations that include severe side effects, unfavorable toxicity profiles, prolonged treatment duration, difficult administration procedures, and development of drug resistance [7,8,9]. The investment in these therapeutic areas by large pharmaceutical companies is not financially attractive due to the poor prospect of financial returns. Thus, the research of drugs against these diseases is not motivated by commercial reasons. Additionally, many pharmaceutical companies take an opportunistic approach to drug repositioning, using drugs that were previously developed and registered for other therapeutic indications and applying them in the treatment of NTDs. This strategy has obvious advantages, namely reduced development costs. However, the major disadvantage is the non-introduction of new specific drugs used in the treatment of these diseases [7].

Therefore, the discovery and development of new drugs is essential and urgent and should embrace the development of new therapeutic classes, the reduction in toxicity in the host, better administration processes, and the development of combined therapies [10]. One of the main strategies includes the phytochemical study of plants and other natural sources (marine organisms, animals, microorganisms, and fungi). Natural products have played an important role in the drug discovery and development processes [11]. However, in recent decades, most pharmaceutical companies have reduced their drug discovery and development programs from natural sources, largely due to the development of combinatorial chemistry programs [7]. Nevertheless, despite the large number of drugs derived from total synthesis, natural products and/or synthetic derivatives using their novel structures, contribute to the global number of new chemical entities that continue to be introduced on the market [12].

Over the last decade, several reviews have reported the bioactivity of natural products against protozoan neglected diseases [7,13,14,15,16,17,18,19,20,21]. However, natural products described in these reviews were obtained from different sources, including microbial [19], endophytes [20] and other fungi [21], marine [22], or animal origins [23,24]. Some reviews also present a mix of natural compounds origins [7,17]. Regarding natural products from plants, since 2016, there have been some reviews focusing exclusively on compounds from higher plants [16,18,25,26,27,28,29,30]. Nevertheless, the information is scattered amongst the diverse antiprotozoal diseases, compound families, and sources. To the best of our knowledge, a comprehensive review gathering the data concerning the most recent studies on terpenic compounds with antitrypanosomal activities is still missing. Therefore, in this work, a compilation of terpenes obtained from plants and evaluated for their activity against *T. brucei* and *T. cruzi*, covering the period from 2016 to 2021, will be presented and discussed. When available, data regarding in vivo activity and considerations about possible mechanisms of action will be also addressed.

The literature search was performed from June to December 2021 using Web of Science, ScienceDirect, PubMed, and some official websites (WHO, DNDi, CDC). An appropriate combination of keywords and truncation was selected and adapted for each database (for example, combinations of terpenoids or terpenes with Trypanosoma, Human African trypanosomiasis, and Chagas disease). Only peer-reviewed research articles or reviews in a six-year timespan (2016–2021) and in English language were considered. No restriction geographical origin of authors was applied. In particular cases, important reviews older than six years were also included. The literature was individually screened, applying as exclusion criteria, poor quality, inaccurate data, not considered relevant to the aim of the review, and articles reporting antitrypanosomal activity of extracts. Mendeley Reference Manager Software (2020) was used to manage the references and eliminate duplicates.

## 2. Trypanosomiasis

### 2.1. Human African Trypanosomiasis (HAT)

**Figure 1:**
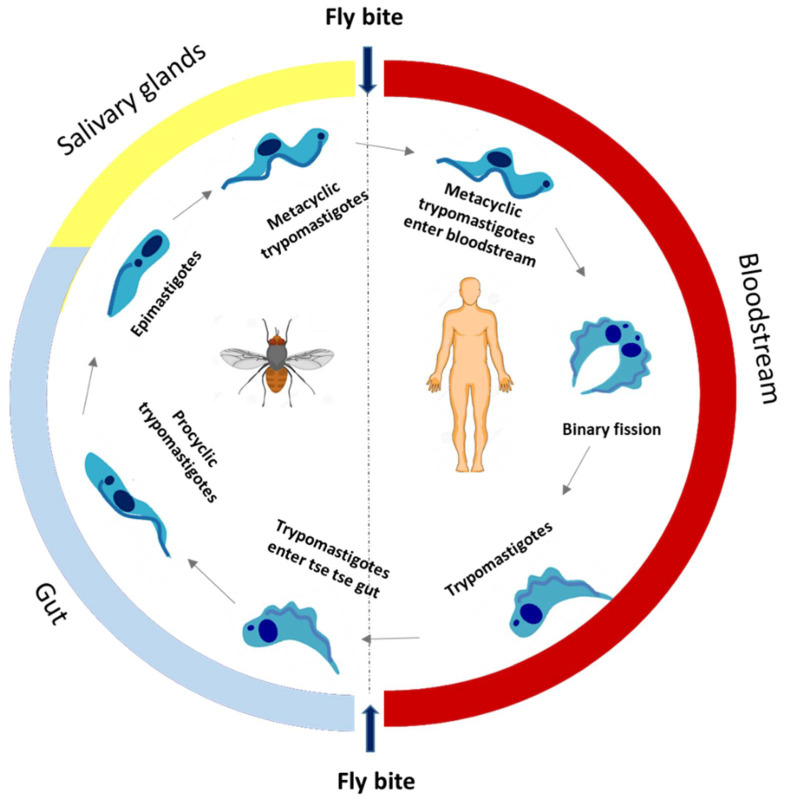
The life cycle of *T. brucei*.

HAT is endemic in 36 African countries, with approximately 60 million people at risk, and approximately 10.8 million people living in areas of moderate to high risk of infection. In 1995, about 25,000 cases were detected and about 300,000 cases remained undetected. However, in 2001, the WHO launched an initiative to strengthen control and surveillance, and HAT declined in the following years. In 2019, less than 1000 cases were reported. It is noteworthy that this reduction is not due to a lack of control efforts as active and passive screening have been maintained at similar levels (about 2.5 million people screened per year) [31]. HAT is essentially present in poorer and rural areas, affecting populations dedicated to agriculture, fishing, livestock, and hunting, who are more exposed to the vector of transmission. Furthermore, their access to adequate health services is limited, thus lacking medical surveillance, also associated with difficulties in diagnosis and treatment [32]. This parasitic disease is transmitted mostly by the bite of the tsetse fly (*Glossina palpalis*), but other routes of transmission are possible, such as congenital transmission, blood transfusion, and transplants, despite being poorly documented [33]. The life cycle of *Trypanosoma brucei* sp. is illustrated in Figure 1 and reviewed elsewhere [10,34].

There are two subspecies of *Trypanosoma brucei* (*T. b*.), *T. b. gambiense*, and *T. b. rhodesiense*, with different geographic distributions. *T. b. gambiense* is found in 24 countries in West and Central Africa, accounting for more than 98% of reported cases. *T. b. rhodesiense* is present in 13 countries in East Africa, representing less than 3% of reported cases [32]. The two subspecies have different rates of progression and clinical characteristics. Infections by *T. b. gambiense* are characterized by a low parasitemia, with slow progression leading to the development of the chronic form of the disease, while *T. b. rhodesiense* progresses rapidly with high parasitemia, being characterized by the acute form. Both infections, if not diagnosed and treated, lead to death [34]. The clinical evolution of HAT has two phases. In the first phase, also known as the hemolymphatic phase, the parasite is found in the host’s blood and lymphatic stream. This initial phase includes non-specific symptoms such as pyrexia, headache, muscle and joint pain, weight loss and even enlarged lymph nodes, usually in the neck area. The second or neurologic phase (meningoencephalitis) occurs when the parasite crosses the blood–brain barrier and reaches the central nervous system. The clinical manifestations are usually behavioral changes, such as anxiety and irritability, sensory, motor, and sleep cycle disorders [32,33].

#### 2.1.1. Antitrypanosomal Chemotherapy Targets and Current Drugs against HAT

Trypanosome-specific metabolic and cellular pathways represent excellent molecular targets. The ability to synthesize polyamines, putrescine, and spermidine, is of vital importance for the proliferation of bloodstream forms in trypanosomes. In this process, ornithine decarboxylase has a crucial function. This enzyme is considered the best-validated drug target in *T. brucei*, which is the target of eflornithine, a drug that is used clinically for the treatment of HAT [10,35]. In addition, the enzyme N-myristoyltransferase (NMT) has been well validated as a molecular target for HAT since its inhibition may lead to the death of the parasites. NMT catalyzes the covalent attachment of myristate, a 14-carbon saturated fatty acid, via amide bond to the N-terminal glycine residue of several proteins. NMT is also present in humans, but *T. brucei* is extremely sensitive to NMT inhibition, probably because endocytosis occurs at a very high rate in *T. brucei* [36]. Recently, significant progress in targeting the ubiquitin-proteasome system was reported [37]. The ubiquitin-proteasome system (UPS) is a crucial protein degradation system in eukaryotes and is essential for the survival of eukaryotes including trypanosomatids. There are promising inhibitors of this, but the overall success of clinical trials is low and therefore more drug candidates are needed. The bloodstream forms of *T. brucei* produce energy exclusively through glycolysis. Thus, inhibition of glycolytic enzymes, such as glyceraldehyde 3-phosphate dehydrogenase, phosphoglycerate mutase, phosphofructokinase and pyruvate kinase, could be a potential therapeutic approach. However, there is little prospect of killing trypanosomes by suppressing glycolysis unless inhibition is irreversible or uncompetitive, owing to the enormous glycolytic flux through the system [10,35]. Regarding redox metabolism, a fundamental metabolic difference between host and parasite is the existence of trypanothione reductase in trypanosomes instead of glutathione reductase, which is essential for the parasite’s survival. The inhibition of trypanothione reductase compromises the parasite’s oxidative defenses, sometimes leading to its death. Unfortunately, until now, compounds suitable for clinical development have not been discovered [10].

Currently, there is no vaccination or chemoprophylaxis for HAT, and its combat is mainly conducted through prophylactic measures aimed at reducing the reservoir of the disease and controlling the vector. The latter is the main strategy in use, which aims to minimize human contact with the fly. The recommended measures in the most affected areas are the use of clothes with neutral colors, and the use of insecticide repellents [34]. Recently, rapid diagnostic tests have been also developed in order to detect the presence of the antigen, offering accurate and sensitive results [38]. The treatment of HAT depends essentially on the stage of the disease and the causative agent. Until recently, five drugs have been used to treat sleeping sickness, donated by manufacturers to WHO for free distribution [38]. For the first phase, suramin (Naganinum^®^, Naganol^®^) and pentamidine (Nebupent^®^, Pentam^®^) are the first line drugs. For the final stage of sleeping sickness, the treatment includes the use of melarsoprol (Arsobal^®^), eflornithine (Vaniqa^®^) and the nifurtimox (Lampit^®^) -eflornithine combination therapy (NECT) [39]. Recently, fexinidazole was approved by the European Medicine Agency (EMA) and the United States Food and Drug Administration (USFDA) as the first all-oral therapy for the treatment of phase-1 and phase-2 HAT [39]. Additionally, acoziborole a recently developed benzoxaborole, is currently in advanced clinical trials, for treatment of phase-1 and phase-2 caused by both *T.b. gambiense* and *T.b. rhodesiense*. Acoziborole is orally bioavailable, and importantly, curative with one dose [40].

### 2.2. Human American Trypanosomiasis (Chagas Disease, CD)

**Figure 2:**
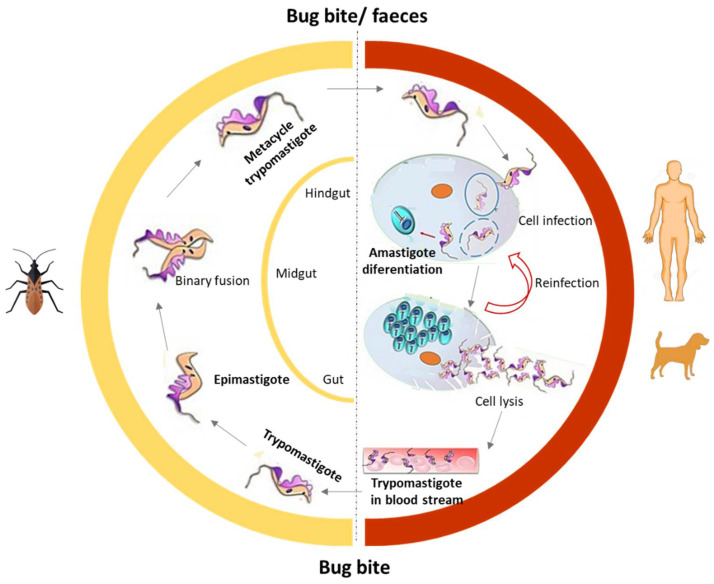
The life cycle of *T. cruzi*.

Human American trypanosomiasis, commonly known as Chagas disease (CD), is endemic in 21 countries in Latin America. However, human migrations have turned it into a global disease with a significant number of cases in non-endemic regions such as Canada or Europe, among others. CD is present in rural areas and affects populations living in poverty. The WHO estimates that about 6 to 7 million people worldwide are infected and there are approximately 70 million people at risk [41,42]. CD is caused by the protozoan parasite *Trypanosoma cruzi* (*T. cruzi*), and it is generally transmitted by vectors, such as the triatomine hematophagous insects of the *Reduviidae* family, usually known as “barbers”. The other transmission routes are blood transfusions, transplantation, congenital transmission, and oral transmission (breast milk), and also by ingestion of contaminated food. The life cycle of *Trypanosoma cruzi* is illustrated in Figure 2 and reviewed elsewhere [41,42,43].

CD has two successive clinical phases, an acute phase, and a chronic phase. The acute phase can be symptomatic or, most frequently asymptomatic. The initial acute phase occurs immediately after the infection, which can last for weeks or months. It is characterized by local manifestations such as the Romaña Sign, when the parasite penetrates the conjunctiva, or the skin, causing a skin lesion or a purplish swelling of the lids of one eye called Chagoma. After a period of 4 to 8 weeks, the parasitemia decreases and the clinical manifestations spontaneously disappear in 90% of the cases, when the disease enters the chronic phase [41,43]. During the chronic phase, the parasites are in the heart and gastrointestinal tract. Despite the long-lasting nature of the infection in which most individuals do not develop overt pathology, there are about 30% of people who can achieve the chronic phase, characterized by progressive heart and/or digestive disease. In most of these cases, it takes decades to become apparent. Cardiomyopathy is the most serious result of *T. cruzi* infection, and in many areas of South America, it is a major cause of heart disease. Digestive symptoms, including megaesophagus and megacolon, also have serious consequences and may require surgery [43,44,45,46,47]

#### 2.2.1. Antitrypanosomal Chemotherapy Targets and Current Drugs against CD

Infective trypomastigotes and intracellular replicative amastigotes are the clinically relevant life-cycle stages of *T. cruzi* that are potential targets for drug intervention [4,48]. *T. cruzi* requires specific sterols for cell viability and proliferation at all stages of the life cycle. The main sterol component of the parasite is ergosterol, while in the mammalian hosts it is cholesterol. Inhibitors of sterol biosynthesis have been shown antitrypanosomal in vitro activity [48]. Other trypanosomal targets are related to cysteine proteases that are involved in many crucial processes, including host cell invasion, cell division, and differentiation. *T. cruzi* contains a cysteine protease, cruzipain, which is responsible for proteolytic activity at all stages of the parasite’s life. Although no inhibitors of this family of enzymes have progressed to clinical trials, the parasite cysteine proteases remain a promising area of research [47,48]. In addition, the trypanothione reductases and synthetases have also been considered key enzymes in the oxidative metabolism of the parasite. Although several potential inhibitors of the trypanothione reductase possess potent in vitro anti-*T. cruzi* activity, to date, none have achieved parasitological cure in animal models [47].

Only two old nitroheterocyclic drugs, benznidazole (Rochagan^®^ or Rodanil^®^) and nifurtimox (Lampit^®^) have been available for the treatment of CD, as reviewed elsewhere [41,49]. They are effective for the acute phase of infection, but they have variable efficacy in the chronic phase of the disease, besides requiring prolonged treatment (60–90 days). In addition, significant problems of resistance have emerged with both drugs. In this context, there is an urgent need for more efficacious and safer drugs or drugs regimens, in particular for the treatment of the chronic stage of the infection. Presently, new benznidazole monotherapy regimens with reduced exposure to improve tolerability while maintaining efficacy, and combination regimens of benznidazole with fosravuconazole to improve efficacy are being developed [50].

## 3. Terpenic Compounds with Antitrypanosomal Activity

**Figure 3:**
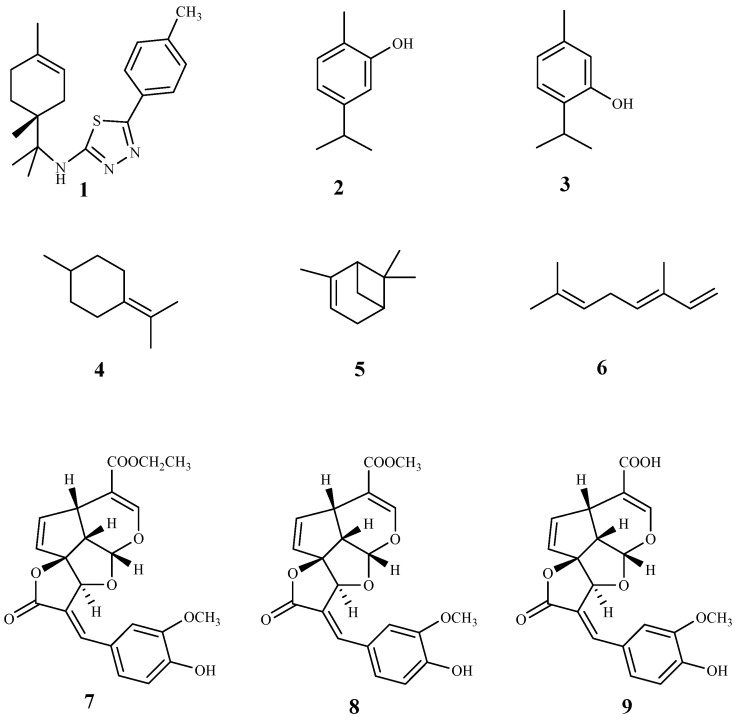
Structures of monoterpenes (**1**–**6**) and iridoids (**7**–**9**).

**Figure 4:**
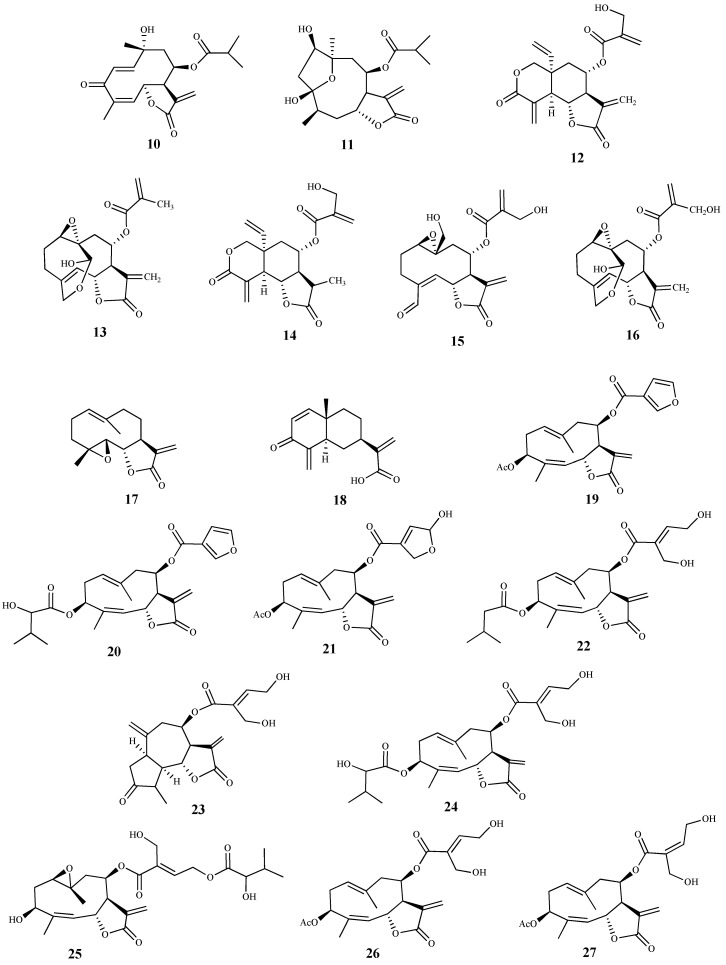
Structures of sesquiterpenes **10**–**27**.

**Figure 5:**
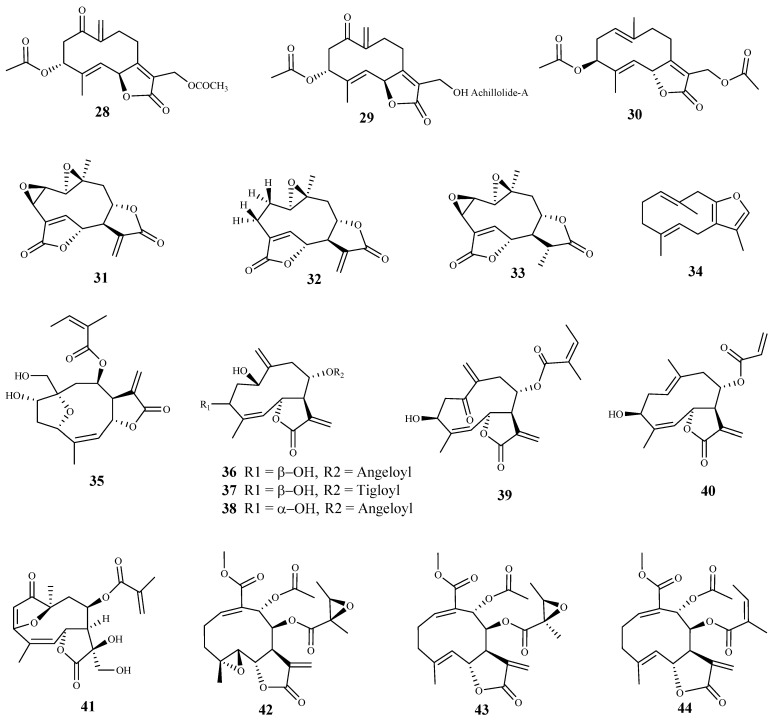
Structures of sesquiterpenes **28**–**44**.

**Figure 6:**
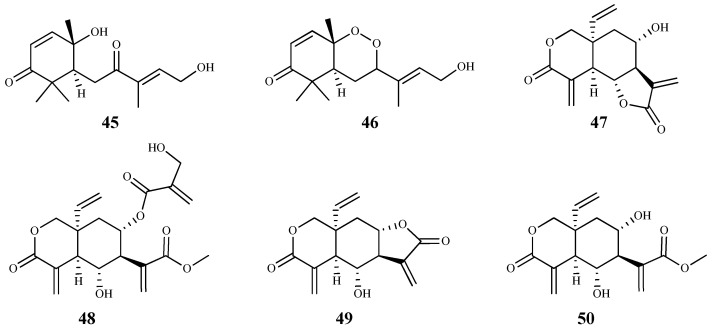
Structures of sesquiterpenes **45**–**50**.

**Figure 7:**
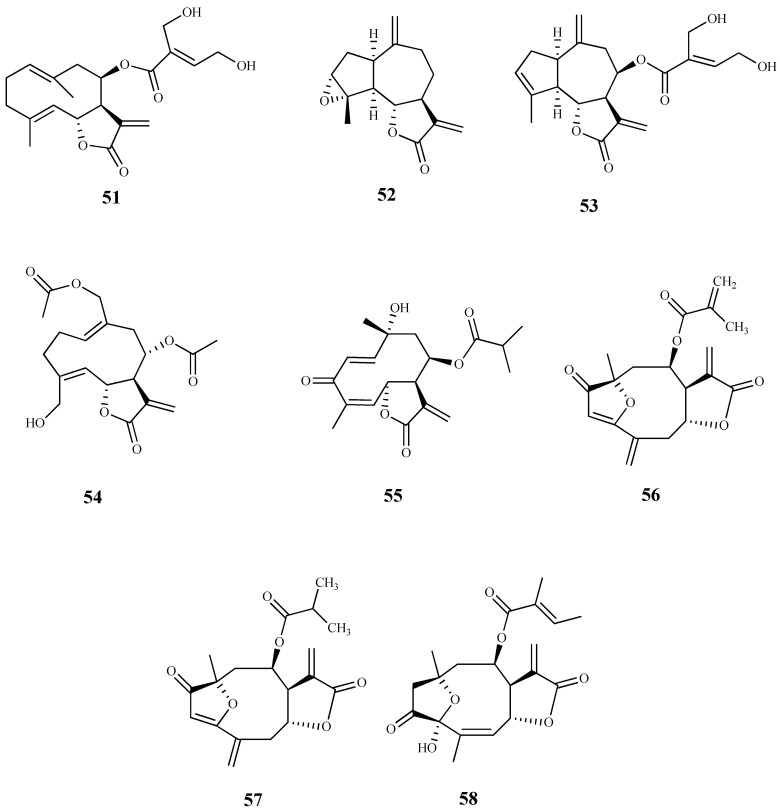
Structures of sesquiterpenes **51**–**58**.

**Figure 8:**
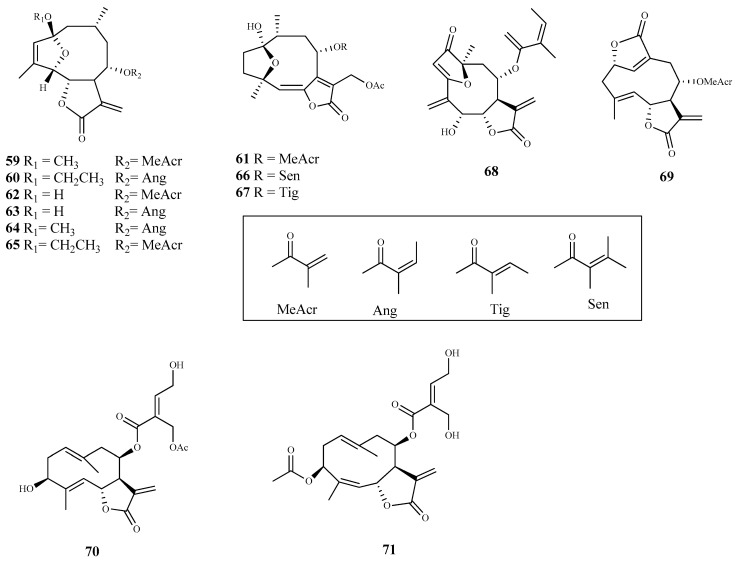
Structures of sesquiterpenes **59**–**71**.

**Figure 9:**
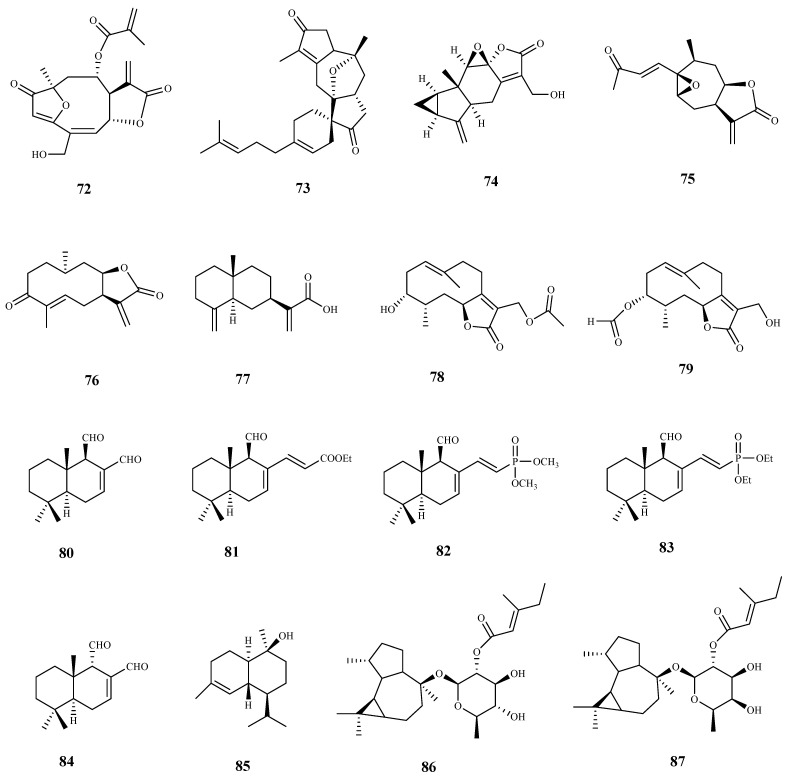
Structures of sesquiterpenes **72**–**87**.

**Figure 10:**
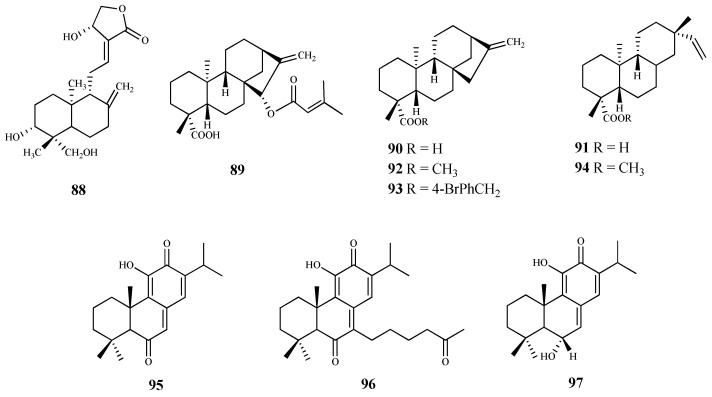
Structures of diterpenes **88**–**97**.

**Figure 11:**
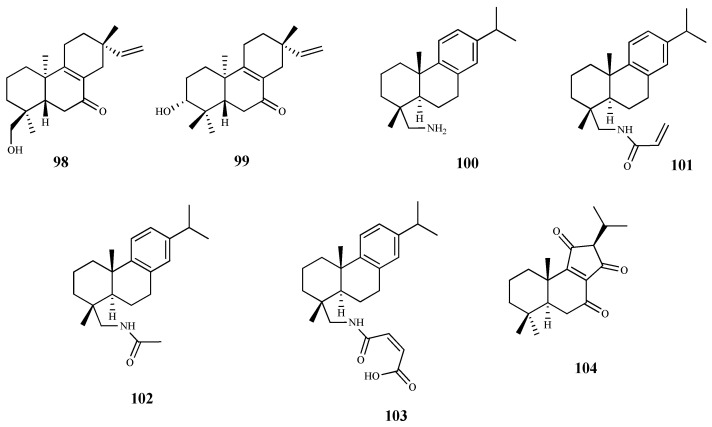
Structures of diterpenes **98**–**104**.

**Figure 12:**
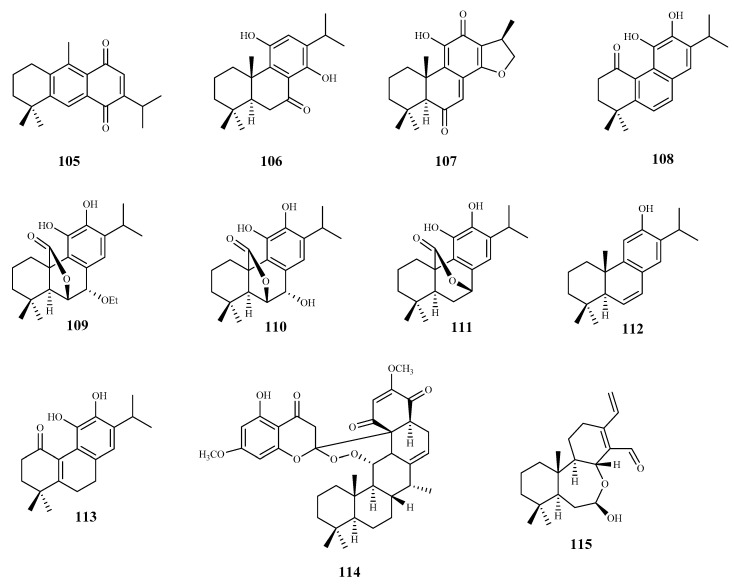
Structures of diterpenes **105**–**115**.

**Figure 13:**
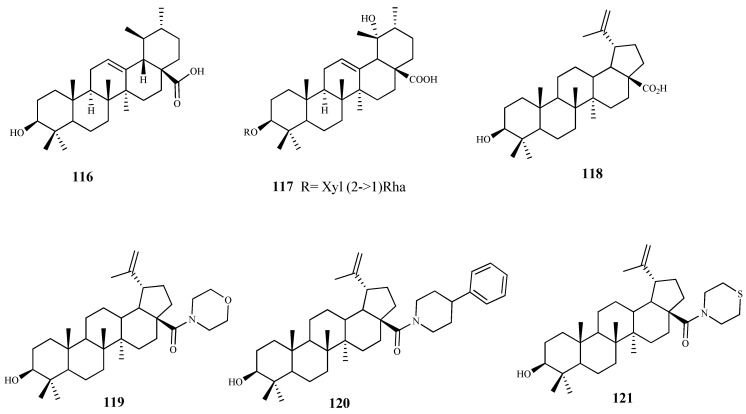
Structures of triterpenes **116**–**121**.

**Figure 14:**
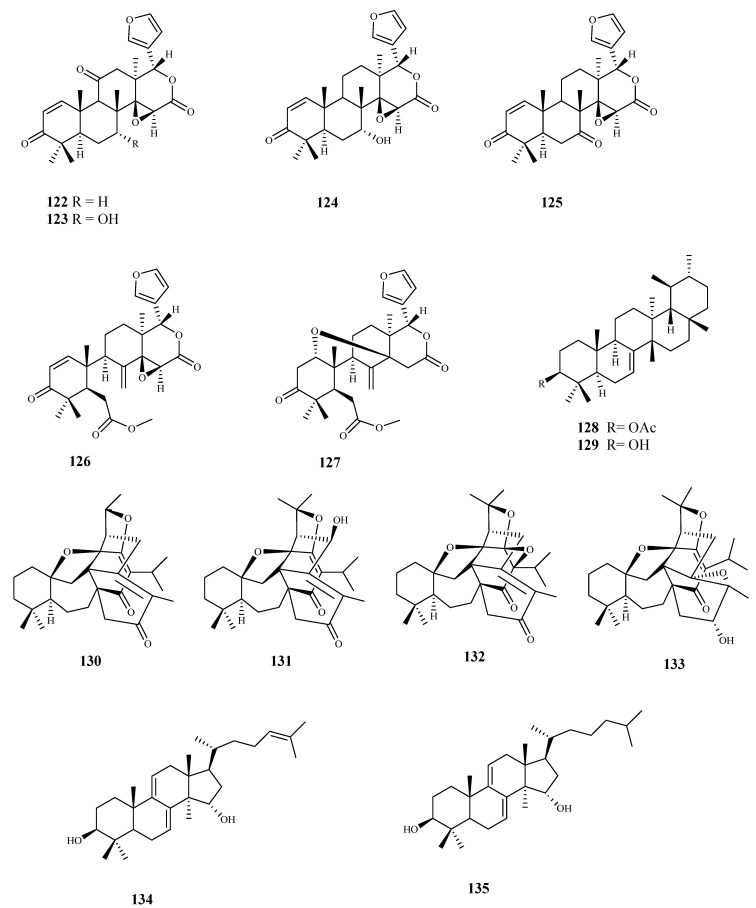
Structures of triterpenes **122**–**135**.

**Figure 15:**
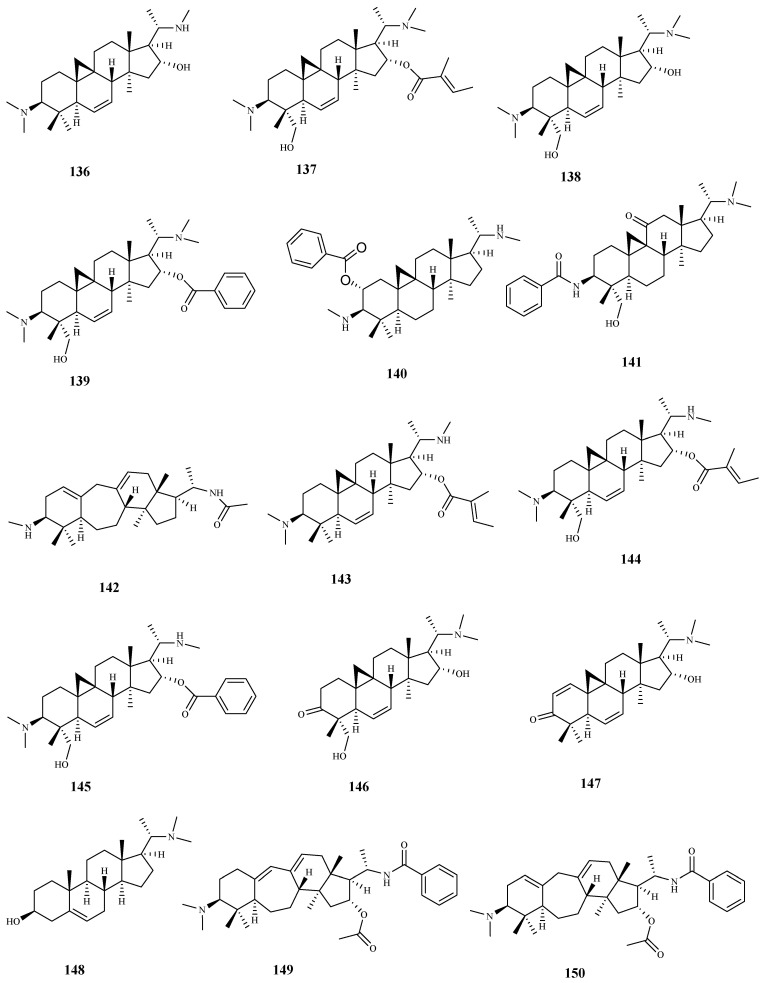
Structures of triterpenes **136**–**150**.

Herein, 150 terpenic compounds with antitrypanosomal activity, isolated from plants or obtained by derivatization, and reported in the literature from 2016 to 2021, are presented (Figure 3, Figure 4, Figure 5, Figure 6, Figure 7, Figure 8, Figure 9, Figure 10, Figure 11, Figure 12, Figure 13, Figure 14 and Figure 15 and Table 1 and Table 2). For clarity reasons, the terpenes are divided into four classes: monoterpenes and iridoids (C10, Figure 3), sesquiterpenes (C15, Figure 4, Figure 5, Figure 6, Figure 7, Figure 8 and Figure 9), diterpenes (C20, Figure 10, Figure 11 and Figure 12), and triterpenes (C30, Figure 13, Figure 14 and Figure 15). The selected compounds were tested for their in vitro activity against *T. brucei* (*T. b. brucei, and T. b. rhodesiense*), and *T. cruzi*. Additionally, the in vivo results of some compounds were also described.

The in vitro antitrypanosomal activities are expressed in micromolar concentrations (µM) and some were transformed into this unit to allow an accurate comparison. Furthermore, the in vitro cytotoxicity of these compounds on mammalian cells lines is also indicated, when evaluated simultaneously, allowing the assessment of the selectivity index (SI). SI is defined as the ratio between the half-maximal cytotoxic concentration against the mammalian cell line (CC_50_) and the half-maximal inhibitory concentration against the parasite (IC_50_) [51,52]. Although the SI values do not allow extrapolation to the in vivo condition, this parameter is valuable for the selection of compounds with selective activity against trypanosomes.

Presently, there are general and specific criteria proposed by DNDi aiming at identifying hit and lead compounds for further development of drugs against trypanosomiasis, and other infectious diseases. Although these criteria are not strictly applied, they are very important to guide the development of hit and lead series, taking into account their potency, selectivity, toxicity, and chemical profile, among other requirements [51]. For a hit definition, the criteria are divided into two main sets: the disease-specific criteria that focus on potency, efficacy, pathogenicity, and the compound-specific criteria that evaluate the chemical profile of the compounds, in silico pharmacokinetics and pharmacodynamics (DMPK), as well as the physical properties that are predictive of oral therapy [52]. Accordingly, a compound is considered active if it has an IC_50_ ≤ 10 µM in the in vitro assay against the bloodstream forms of *T. b. brucei* subspecies, and against the *T. cruzi* intracellular amastigote forms (TcVI (Tulahuen) or TcII/Y strain) [51,52]. The selectivity of the promising hit compound should be 10-fold higher for the parasite than for the mammalian cell line tested. On the other hand, a lead compound for a future drug against HAT or CD should display an IC_50_ value more than 10–20-fold higher than the IC_50_ value of the hit compound, and ideally, its selectivity should be ≥ 50 times higher for the parasite than for the mammalian cell line. Moreover, a significant reduction in parasitemia and/or increase in life-span should be observed in the acute mouse model of HAT at the end of the treatment with up to 4 doses at 50 mg/kg (*i.p* or *p.o)*. Concerning CD, the lead selection criteria include a hit that causes an 80% parasitemia reduction in organs or tissues, or no parasites detected at the end of treatment and an increase in lifespan with up to 10 doses at 50 mg/kg (*p.o*) in a mouse model [51,52].

### 3.1. Monoterpenes and Iridoids

Compound **1** is a limonene benzaldehyde-thiosemicarbazone derivative that showed in vitro antitrypanosomal activity and high selectivity against *T. cruzi* amastigotes (IC_50_ 1.3 µM, CC_50_ 795 µM, mammalian LLCMK2; SI = 611.2). It is believed that this compound act by inhibiting the proliferation of *T. cruzi* and inducing morphological changes that lead to the cell death of the parasite. In addition, a reduction in cell volume, depolarization of the mitochondrial membrane and an increase in production of reactive oxygen species (ROS) were also observed. Due to promising in vitro results, an in vivo study was performed on a murine model of acute Chagas disease, and a significant reduction in parasitemia in animals treated with **1** alone (100 mg/kg/day) or combined with benznidazole (5 mg/kg/day each) was found, when compared to the untreated animals. Moreover, it was observed that the survival rate of the animals treated with both compounds during the period of infection was the same that the group treated just with benznidazole, however, with only 5% of the dose used [53].

The essential oil of *Origanum onites* L. and its major components carvacrol (**2**) and thymol (**3**) were evaluated for their antitrypanosomal activity against *T. b. rhodesiense* trypomastigote forms (mammalian stage), and *T. cruzi* amastigotes. Good results were only observed against *T. b. rhodesiense*, and both compounds showed IC_50_ values of 1.0 µM and 0.73 µM, respectively, and a high selectivity for the parasite (SI = 327.5 and 454.4, respectively, L6 mammalian cell line). Additionally, in the in vivo *T. b. brucei* mouse model, only compound **3** extended the mean survival of animals, while none cured the infected animals when compared to the reference drug pentamidine [54].

The essential oils of some Apiaceae plants (*Echinophora spinosa* L., *Sison amomum* L., *Crithmum maritimum* L., *Helosciadium nodiflorum* (L.) W.D.J.Koch) were studied against *T. brucei* bloodstream forms (TC221 BSFs strain), showing IC_50_ values in the range of 2.7–10.7 µg/mL. From those, only the essential oil of *C. maritimum* had a good selectivity (SI = 13, mouse Balb3T3 fibroblasts cell line). Additionally, using the same parasite, the trypanocidal activity of the major compounds (**4**–**6**) of *C. maritimum* was also tested. Terpinolene (**4**) was the most potent showing an IC_50_ value of 0.26 µM with a SI = 180. Two other compounds displayed promising activities on the same model namely α-pinene (**5**, IC_50_ = 7.4 µM, SI > 100) and β-ocimene (**6**, IC_50_ = 8 µM, SI > 91) [55].

Three tetracyclic iridoids (**7**–**9**) were isolated from *Morinda lucida* Benth., a plant traditionally used to treat parasitic diseases in West Africa. Iridoids were evaluated for their in vitro activity against the bloodstream forms of *T*. *b. brucei*. Compound **7** was the most active (IC_50_ 0.43 μM) and less toxic than **8** (IC_50_ 1.27 μM), displaying CC_50_ values of 14.24 μM (SI = 33.1) and 4.74 μM (SI = 3.7), respectively [56]. The activity of compound **9** is lower than **7** and **8** (IC_50_ 3.75 μM), but it did not show cytotoxicity (CC_50_ ≤ 50 µM) [56]. The main structural differences between compounds **7**–**9** are the functional groups at C-4 on the side chain. Compound **9** has a carboxylic acid, while compound **7** and **8** have ethyl ester and methyl functional groups, respectively [56]. SI values of the three compounds showed that **7** and **9** are more specific against the parasite than compound **8**. Compound **7** was tested in vivo, and a complete clearance of parasitemia, with 100% cure for 20 days post infection, was observed, when 5 consecutive daily shots of 30 mg/kg of compound **7** were taken. It was concluded that compounds **7** and **9** suppressed the expression of paraflagellum rod protein subunit 2, and caused cell cycle alteration, which can preceded apoptosis induction in the bloodstream form of the parasite [56].

### 3.2. Sesquiterpenes

Two sesquiterpene lactones (**10** and **11**) were isolated from the methanolic extract of *Tithonia diversifolia* (Hemsl) A. Grey, and their activities were evaluated against the blood forms of T. brucei [57]. Compound **10** was the most active, exhibiting a very low IC_50_ value (0.012 μM), but displaying a high cytotoxicity on the mammalian fibroblasts cells (CC_50_ 0.036 μM, SI = 3). Likewise, compound **11** showed antitrypanosomal activity (IC_50_ 0.97 μM) and high cytotoxicity (CC_50_ 1.27 μM, SI = 1.3) [57]. Some considerations can be made regarding the mechanism of action of these sesquiterpene lactones in the parasite cells. Due to the characteristic α,β-unsaturated lactone function present in these structures, which can act as a Michael acceptor, these compounds react with nucleophiles, such as thiol groups in proteins, leading to macromolecular dysfunction, oxidative stress and genetic mutations [57]. The presence of an extra carbonyl group conjugated with two double bonds in **10** can explain the higher antitrypanosomal activity of **10** when compared with **11**. In fact, the mechanism of action of these compounds against trypanosomes may be related with formation of thiol adducts with components found in the intracellular medium (namely trypanothione, glutathione and thiol groups in proteins). The parasite’s cells become more vulnerable to oxidative stress with reduction in trypanothione [57].

Several sesquiterpene lactones were isolated from the dichloromethane extract of *Vernonia cinerascens* Sch.Bip., and evaluated for their in vitro activity against the blood forms of *T. b. rhodesiense* and for cytotoxicity on the L6 mammalian cell line [58]. Lactone **12** was the most active and selective exhibiting an IC_50_ value of 0.16 µM and SI = 35. Compounds **13** and **14** also showed activity against *T. b rhodesiense* (IC_50_ values of 0.5 µM and 1.1 µM, SI = 13 and 4.2, respectively), being lactone **13** the most selective [58]. Compounds **15** and **16** exhibited similar activities; however, compound **15** showed a higher selectivity (IC_50_ values of 4.8 µM and 5.0 µM, SI = 27 and 4.3, respectively). Moreover, lactones **12**–**16** displayed the lowest cytotoxicity in the cells tested [CC_50_ 5.6 µM (**12**), CC_50_ 6.9 µM (**13**), CC_50_ 4.7 µM (**14**), CC_50_ 128 µM (**15**), CC_50_ 22 µM (**16**)] [58,59].

Two sesquiterpene lactones (**17** and **18**) isolated from the dichloromethane extract of *Tarchonanthus camphoratus* L. aerial parts, and twenty sesquiterpene lactones, including compounds **19**–**27** obtained from *Schkuhria pinnata* (Lam.) Kuntze ex Thell. were studied for their in vitro antitrypanosomal activity and cytotoxicity on mammalian L6 cell lines [60]. Lactones **17** and **18** were active against *T. b. rhodesiense* with IC_50_ values of 0.39 μM and 2.8 μM, respectively. Furthermore, **17** (SI = 18.6, CC_50_ 7.2 μM) proved to be more selective although it was more cytotoxic than **18** (SI = 6.2, CC_50_ 17.3 μM) [60]. Regarding compounds isolated from *S. pinnata*, most of them displayed antitrypanosomal activity with IC_50_ values ranging from 0.10 to 7.30 μM, Compounds **19** and **20** stood out for their high activities against the trypomastigotes, with IC_50_ values of 0.10 and 0.13 μM, respectively. However, they exhibited cytotoxicity on the cells tested (CC_50_ values of 2.10 and 3.90 μM, respectively) despite exhibiting some selectivity (SI = 20.5 and 29.7, respectively). Compounds **21** (IC_50_ 0.35 μM, SI = 11.5) and **22** (IC_50_ 0.52 μM, SI = 13) were particularly active, but cytotoxic against the mammalian cell lines assayed (CC_50_ values of 4.10 and 6.80 μM, respectively). Moreover, compounds **23** (IC_50_ 0.60 μM, SI = 19.2), **24** (IC_50_ 0.82 μM, SI = 13.4), **25** (IC_50_ 0.91 μM, SI = 15.8) and **26** (IC_50_ 0.92 μM, SI = 15.8) also showed very good activities. Finally, sesquiterpene **27** display an IC_50_ value of 1.7 μM, and was the most selective and least cytotoxic in this group of compounds (SI = 31.1 and CC_50_ 54.6 μM) [60].

Three sesquiterpene lactones (**28***–***30**) were isolated from the dichloromethane extract of *Achillea fragrantissima* (Forssk.) Sch.Bip. and tested against trypomastigote forms of *T. b. brucei* [61]. Lactone **28** was the most active (IC_50_ 3.03 μM), while lactones **29** and **30** showed the same activity against the parasite with IC_50_ value of 10.97 μM. The authors did not assess the cytotoxicity of these compounds on mammalian cells [61].

Four sesquiterpene lactones were isolated by bioassay-guided fractionation from extracts of *Mikania variifolia* Hieron. and *Mikania micrantha* Kunth, and evaluated against the epimastigote, trypomastigote and amastigote forms of *T. cruzi*. Compounds **31** and **32** were found in both extracts (2.2% and 0.4% for *M. variifolia*, and 21.0% and 6.4% for *M. micrantha*, respectively, calculated based on the dry extract) [62]. Three of the isolated lactones (**31**, **32**, and **33**) showed trypanocidal activity, being active against the epimastigote form with IC_50_ values of 2.41 (SI = 31.9), 0.29 (SI = 992.5) and 8.55 (SI = 5.2) μM, respectively [62]. Compounds **31**, **32** and **33** also displayed activity against the trypomastigote form of the parasite with IC_50_ values of 7.24 (SI = 10.6), 5.43 (SI = 54.0) and 1.03 (SI = 49.0) μM, respectively. Finally, the activities of **31**, **32** and **33** against the amastigote forms were lower than those observed for the two previous forms of the parasite (IC_50_ 15.5, 22.8 and 29.1 μM, and SI = 4.3, 12.5, and 1.5, respectively). From those compounds, **32** was the most selective for the human infective parasite, showing a SI of 54 when assayed on human monocyte leukemia THP1 cells. Due to its good selectivity, **32** was also tested in an in vivo model of *T. cruzi* infection, and was able to decrease the parasitemia and the weight loss associated with the acute phase of the parasite infection. Additionally, 70% of treated mice (1 mg/kg of body weight/day) survived, while all of the control mice died by day 22 after the infection. The authors also observed that this compound increased the production of TNF-*α* and IL-12 by macrophages [62].

The essential oils from different parts of *Smyrnium olusatrum* L. were evaluated against the bloodstream forms of *T. b. brucei*. All oils effectively inhibited the growth of the parasite [63]. From the main constituents of essential oils, sesquiterpene **34** exhibited a significant and selective inhibitory activity against the tested parasite (IC_50_ 3.0 µM, SI = 30, mouse Balb/3T3 fibroblast)) [63].

From *Anthemis nobilis* L. dichlorometane extract, 19 sesquiterpene lactones, including 15 germacranolides, 2 seco-sesquiterpenes, 1 guaianolide sesquiterpene lactone, and 1 cadinane acid were obtained [64]. Among these compounds, thirteen were tested for their in vitro activity against the bloodstream forms of *T. b rhodesiense*, with compound **35** being the most potent and selective (IC_50_ 0.08 μM, SI = 63.1). Compounds **36**–**38** exhibited also a significant anti-trypanosomal activity, but with lower selectivity (**36**, IC_50_ 0.61 μM, SI = 8.3; **37**, IC_50_ 0.36 μM, SI = 14.1; **38**, IC_50_ 0.88 μM, SI = 8.3). Moreover, the compounds were assessed against *T. cruzi* intracellular amastigotes, and the best result was observed for compound **39** (IC_50_ 2.8 μM), but a very low selectivity index was also observed (SI = 0.5). Compound **39** also exhibited a good activity against *T. b rhodesiense* (IC_50_ 0.4 μM), but with a low selectivity due to its cytotoxicity on mammalian L6 cells (CC_50_ 1.5 μM, SI = 3.8). Compound **40** was considered the one with higher selectivity for *T. cruzi* (IC_50_ 4.2 μM, SI = 6.1) [64].

*Calea pinnatifida* (R. Br.) Less. is used in folk medicine as giardicidal, amoebicidal and to treat digestive disorders. Its phytochemical study led to the isolation of a furanoheliangolide sesquiterpene lactone (11,13-dihydroxy-calaxin, **41**) which showed a promising trypanocidal activity, displaying an IC_50_ value of 8.30 μM against *T. cruzi* amastigotes, and inhibiting the parasite growth in 94.3%. However, compound **41** presented a low selectivity for the parasite cells (CC_50_ < 15.60 μM on THP-1 cells) [65].

Three sesquiterpene lactones (**42***–***44**) were isolated from *Smallanthus sonchifolius* (Poepp.) H. Rob. and evaluated against *T. cruzi* epimastigotes, using benzonidazole as positive control [66]. Compounds **42** and **43** showed identical activities with IC_50_ values of 0.78 and 0.79 μM, respectively. Lactone **44** was also active exhibiting an IC_50_ value of 1.38 μM. All compounds were more effective than benzonidazole (IC_50_ 10.6 μM). The authors did not assess the cytotoxicity of these compounds on mammalian cells. Due to the high in vitro activity, compounds **42** and **43** were also tested on mice inoculated with *T. cruzi* trypomastigotes. A significant decrease in circulating parasites (50–71%) was observed, with no signs of toxicity in the dose administrated (1 mg/kg/day). Complementary studies showed marked ultrastructural alteration in trypanosome parasites when treated with these compounds [66].

Several sesquiterpenes isolated from the Cameroonian spice *Scleria striatinux* De Wild. were studied for their in vitro and in silico antiparasitic activity [67]. From those, sesquiterpene **45** exhibited the best activity against *T. cruzi* and *T. b. rhodesiense* bloodstreams forms, with IC_50_ values of 0.025 μM (SI = 0.74) and 0.002 μM (SI = 8.3), respectively, but the compounds were cytotoxic on HT-29 (human bladder carcinoma) cells. On the other hand, compound **46** showed better activity against *T. b. rhodesiense* than *T. cruzi*, with IC_50_ values of 0.025 μM (SI = 3.4) and 0.085 μM (SI = 1), respectively [67]. The in silico drug metabolism and pharmacokinetic parameters of these two sesquiterpene isomers were also studied, showing compound **46** a good solubility profile, moderate partition coefficient and acceptable in silico pharmacokinetic properties. Similar characteristics were observed for compound **45**, but with less optimal parameters, namely for the partition coefficient [67]. Nevertheless, despite the good pharmacokinetic features and the low IC_50_ values observed, SI values were very small, and compounds also showed a considerable cytotoxicity against HT-29 cell line, which reduces its possible application as a hit compound.

*Vernonia lasiopus* (O.Hoffm.) H.Rob. extracts were obtained with solvents of different polarities and evaluated in vitro for antiprotozoal activity [59]. The dichloromethane extract was shown to be particularly active against *T. b. rhodesiense*, and its phytochemical study led to the isolation and identification of six sesquiterpene lactones. These compounds were tested for their in vitro antitrypanosomal activity and cytotoxicity on L6 mammalian cells [59]. Compound **47**, the main component of the extract, was the most potent against *T. b. rhodesiense* trypomastigotes (IC_50_ 0.185 µM); however, it displayed some cytotoxicity (CC_50_ 2.68 µM and SI = 14.5). Moreover, compound **48** presented very similar values (IC_50_ 0.26 µM, CC_50_ 3.67 µM, SI = 14.4). Lactone **49** was the least selective (SI = 4.5) and displayed some cytotoxicity (CC_50_ 2.26 µM), despite showing a considerable antitrypanosomal activity (IC_50_ 0.51 µM). Compound **50** was the least cytotoxic compound in this group (CC_50_ 34.6 µM, SI = 13.7) still showing a good activity against trypomastigotes (IC_50_ 2.53 µM) [59].

On a recent work, the sesquiterpene lactones eupatoriopicrin (**51**), estafietin (**52**), eupahakonenin B (**53**) and minimolide (**54**) isolated from Argentinean Astearaceae species, which had previously showed activity against *T. cruzi* epimastigotes, were tested against other forms of the parasite [68]. On the bloodstream forms of *T. cruzi* the IC_50_ values obtained were 19.9 µM (**51**, SI = 12.9), 33.0 µM (**53**, SI = 10.4), and 21.0 µM (**54**, SI = 12.8). On the intracellular *T. cruzi* amastigotes the most active compound was **51** with an IC_50_ value of 6.3 µM (SI = 40.6). Moderate activities were observed for compound **54** (IC_50_ = 25.1 µM; SI = 10.7), and **53** (IC_50_ = 89.3 µM; SI = 3.8) against the same form of the parasite. The majority of compounds showed a significant selectivity for the parasite forms tested compared to Vero cells. The in vivo administration of eupatoriopicrin (**51**, 1 mg/kg/day) to mice infected with *T. cruzi* trypomastigotes, for five consecutive days, produced a significant reduction in the parasitemia levels in comparison with non-treated animals (area under parasitemia curves 4.48 vs. 30.47, respectively), being this reduction similar to that achieved with the reference drug, benznidazole. Authors also presented some information regarding the prevention of tissue damage during the chronic phase of the parasite infection, showing beneficial effects on skeletal and cardiac muscular tissues of infected mice treated with the sesquiterpenoid compound. Compound **52** was inactive [68].

The sesquiterpene lactone **55** (tagitinin C) isolated from leaves of *Tithonia diversifolia* (Hemsl.) A. Gray showed a high inhibition activity against the epimastigote forms of *T. cruzi*, with IC_50_ of 1.15 μM, being more active than benznidazole (35.81 μM). However, the cytotoxic concentration (6.54 μM) and the selectivity index (5.69) of compound **55** did not show to be favorable. In an in vivo combination assay, it was observed a complete suppression of parasitemia and parasitological cure in all infected mice (100%) compared to those receiving benznidazole alone (70%). Moreover, despite its lower in vitro selectivity index, compound **55** was well tolerated during the in vivo assays. Interestingly, it was also found that tagitinin C was able to reduce myocarditis, especially when combined with benznidazole [69].

The antitrypanosomal potential of three sesquiterpene lactones (**56**–**58**) isolated from *Helianthus tuberosus* L. (Asteraceae) was evaluated against *T. b. rhodesiense* trypamastigote bloodstream form (**56**, IC_50_ 0.077 μM; **57**, 0.26 μM; **58**, 0.92 μM) and *T. cruzi* trypomastigotes (**56**, IC_50_ 1.6 μM; **57**, 3.1 μM; **58**, 5.7 μM); however, the selectivity index was not promising (CC_50_ between 0.52 and 3.9 μM, on L6 rat skeletal myoblasts) [70].

Seventeen sesquiterpene lactones were isolated from five plant species of Vernonieae tribe and assessed against *T. cruzi* epimastigotes [71]. The best trypanocidal effect was observed by elephantopus-type sesquiterpene lactones **59** (IC_50_ 1.5 µM) and **60** (IC_50_ 2.1 µM), obtained from *Vernonanthura nebularum* (Cabrera) H. Rob., and hirsutinolide **61** (IC_50_ 2.0 µM), isolated from *Vernonanthura pinguis* (Griseb.) H.Rob. Furthermore, these compounds showed a high selectivity for the parasite (SI > 14) when compared to their cytotoxic effect against the mammalian Hela cells. Compounds **62**–**65**, also isolated from *V. nebularum*, showed a good antitrypanosomal activity on the same strain with IC_50_ values ranging from 3.7 to 9.7 µM, being compound **62** the most selective (IC_50_ = 3.7 µM and SI = 14.3). From *V. pinguis*, besides compound **61,** compounds **66** (IC_50_ 10.7 µM; SI = 9.0) and **67** (IC_50_ 8.1 µM; SI = 13.9) were also isolated and displayed a significant activity; however, it was lower than the observed to hirsutinolide (**61**). From the remaining species, compound **68** (IC_50_ 6.8 µM; SI = 1.6) isolated from *Centratherum puctatum* ssp. Punctatum Cass. and compound **69** (IC_50_ 4.7 µM; SI = 11.5) isolated from *Elephantopus mollis* Kunth also showed antitrypanosomal activity [71].

The sesquiterpene lactones eucannabinolide (**70**) and santhemoidin C (**71**), isolated from the dewaxed dichloromethane extract of *Urolepis hecatantha* (DC.) R.King & H.Rob., were active on *T. cruzi* epimastigotes with IC_50_ values of 10 µM and 18 µM, respectively. Both compounds showed low SI values (CC_50_ > 15 µM for **70** and CC_50_ = 15 µM for **71**) [72].

Goyazensolide (**72**) is a sesquiterpene lactone isolated from *Lychnophora passerina* (Mart ex DC) Gardn. that displayed promising results against the intracellular amastigote form of *T. cruzi* (IC_50_ = 0.181 µM/24 h, and IC_50_ = 0.020 µM/48 h), showing a higher selectivity index than the positive control benznidazol (SI = 52.82 and 915.0 for **72**, at 24 h and 48 h, respectively, and SI = 4.85 and 41.0 for benznidazol, at 24 h and 48 h, respectively). Further in vivo assays were performed and **72** showed an important therapeutic activity in mice infected with *T. cruzi*, which was demonstrated by the high percentage of negative parasitological tests employed by the authors in the successive post-treatment evaluations [73].

From the leaves of *Hedyosmum brasiliense* Mart. Ex Miq., five sesquiterpene lactones together with a sesterpene were isolated and tested against the amastigote and trypomastigote forms of *T. cruzi*. Among the assessed compounds, compound **73**, with a rare terpenoid structure, was the most active displaying an IC_50_ value of 21.6 μM and SI > 9 for the amastigote form, and an IC_50_ value of 28.1 μM and SI > 7 for the trypomastigote. The remaining compounds were inactive, excepting compound **74** that exhibited a very weak activity against both parasite forms tested, and a decrease in selectivity for the parasite when compared with selectivity of compound **73** [74].

The bio-guided fractionation of ethanolic extract of leaves of *Inula viscosa* (L.) Greuter (Asteraceae) led to the isolation of two sesquiterpenoids (**75** and **76**), which were tested against *T. cruzi* epimastigotes with IC_50_ values of 4.99 µM and 15.52 µM, respectively. Both compounds showed modest SI (3.67 and 3.38, respectively), when compared to murine macrophages cells [75]. A preliminary structure-activity study of these compounds demonstrated the importance of the lactone ring to the antiparasitic activity. Regarding the mechanism of action, authors suggested that compounds induced programmed cell death in the tested parasite [75].

Costic acid (**77**), a eudesmane sesquiterpenoid isolated from the bio-guided fractionation of the n-hexane extract of *Nectandra barbellata* Coe-Teix. Twigs (Lauraceae) induced a trypanocidal effect with high selectivity for the intracellular amastigote form of *T. cruzi* (IC_50_ 7.9 µM). A modest activity against *T. cruzi* trypomastigotes was also observed (IC_50_ 37.8 µM). No cytotoxicity was observed on L929 human cells, revealing its selectivity for both forms of the parasite (CC_50_ > 200 µM, SI > 25 on amastigote forms and SI > 5 on trypomastigote forms). The authors suggested that costic acid (**77**) has a key action on the mitochondria activity of the parasite [76].

Some germacranolide sesquiterpene lactones were isolated from the aerial parts and flowers of *Tanacetum sonbolii* Mozaff. Compounds **78** and **79** were the most active showing an IC_50_ of 5.1 and 10.2 µM, respectively, against *T. b. rhodesiense* bloodstream forms, and SI values of 3.9 (**78**) and 4.0 (**79**) when compared with rat myoblast (L6) cells [77].

The bicyclic drimane-type sesquiterpene polygodial (**80**), firstly isolated from *Polygonum hydropiper* L. (Polygonaceae), and some natural and synthetic compounds of the same family were evaluated for growth inhibition against the amastigote, trypomastigote, and epimastigote forms of *T. cruzi*. The parent drug **80** exhibited a moderate inhibitory activity (GI_50_ = 34.4 μM amastigotes; GI_50_ = 68.2 μM trypomastigotes; GI_50_ = 51.0 μM epimastigotes). The best inhibition growth activities were observed for its synthetic derivatives, namely compound **81** (GI_50_ = 9.9 μM amastigotes; GI_50_ = 8.4 μM trypomastigotes; GI_50_ = 13.0 μM epimastigotes), **82** (GI_50_ = 6.7 μM amastigotes; GI_50_ = 6.4 μM trypomastigotes; GI_50_ = 12.3 μM epimastigotes), and **83** (GI_50_ = 8.3 μM amastigotes; GI_50_ = 6.9 μM trypomastigotes; GI_50_ = 7.2 μM epimastigotes). Selectivity index values were not determined. The synthetic *α*,β-unsaturated phosphonate (**83**) was favorably compared with the clinically approved drugs benznidazole and nifurtimox during a competition assay, being even effective against trypomastigotes, contrarily to benznidazole that showed no activity against this trypanosomal form. The effect of polygodial derivative **81** on the growth of the parasite in infected human retinal pigment epithelial (ARPE) cells was studied using confocal microscopy. A significant reduction in the intracellular parasites was observed, with no alterations of replication or viability of the cells [78]. Compound **80** was also previously isolated from the Chilean species *Drimys winteri*, and was tested on the same parasite forms with weak comparable results. On this work, the authors associated the trypanosomal activity of this compound with intracellular effects occurring in the parasite, namely, mitochondrial dysfunctions, ROS production and autophagic phenotype [79].

*Epi*-polygodial (**84**), isolated from the Brazilian plant *Drimys brasiliensis* Miers (Winteraceae), exhibited a high parasite selectivity towards *T. cruzi* trypomastigotes (IC_50_ = 5.01 μM, SI > 40 to NCTC cells). Authors correlated the antitrypanosomal activity of this compound with its effects on cellular membranes by the interaction of **84** with DPPE-monolayers (the Langmuir monolayers of dipalmitoylphosphoethanolamine) at the air–water interface, which affects the physical chemical properties of the mixed film [80].

The sesquiterpene (-)-T-cadinol (**85**) isolated from *Casearia sylvestris* Sw. displayed a moderate activity against amastigotes and trypomastigotes forms of *T. cruzi* with IC_50_ values of 15.8 and 18.2 μM, respectively, and no toxic effect on the mammalian cells was observed (CC_50_ 200 μM, SI > 15). The mechanism of action was studied using different techniques, and it was observed that **85** affected the parasite mitochondria. However, additional studies are necessary in order to confirm this organelle as a candidate target [81].

A sesquiterpene glycoside ester (**86**) isolated from the flowers of *Calendula officinalis* L. have shown a moderate activity against *T. brucei* (IC_50_ 16.9 μM). A closely similar compound (**87**), only differing in the type of sugar residue, did not display antitrypanosomal activity, suggesting the importance of sugar moiety conformation to the activity [82].

### 3.3. Diterpenes

Andrographolide (**88**) is a labdane-type diterpene, isolated from *Andrographis paniculate* (Burm. F.) Wall. Ex Nees, with reported anticancer, anti-inflammatory, antioxidant, cardioprotective and hepatoprotective properties [83]. To determine its effect on the viability of *T. brucei* procyclic trypomastigotes (the form of the parasite that differentiate in the insect gut), the parasites were incubated at different concentrations (0–200 μM) for 72 h. Compound **88** inhibited the growth of the parasite, exhibiting an IC_50_ = 8.3 μM and SI = 8.5. At this concentration, no cytotoxic effect was observed (CC_50_ 70.5 μM) [83]. Giemsa staining of parasites treated with **88** allowed the observation of morphological changes, in particular, loss of integrity, damage to the cell membrane, general rounding, and loss of cells’ flagella. Ultimately, the authors concluded that the trypanocidal activity of **88** is mediated by inducing the oxidative stress together with the depolarization of the mitochondrial membrane potential, generating an apoptosis-like programmed cell death [83].

The phytochemical study of aerial parts of *Baccharis retusa* DC., a medicinal plant used in Brazilian folk medicine to treat parasitic diseases, allowed the isolation and identification of the kaurane-type diterpene **89**. This compound was active against *T. cruzi* trypomastigotes (IC_50_ 3.8 μM) with a high selectivity (SI = 50.0) due to its reduced cytotoxicity (CC_50_ 189.7 μM) on NCTC cells [84].

*Ent*-kaurenoic acid (**90**) and *ent*-pimaradienoic acid (**91**) were used as starting material to obtain several derivatives. From those, the *ent*-kaurane derivatives **92** (IC_50_ < 12.5 μM) and **93** (IC_50_ 26.1 μM) showed the highest antitrypanosomal activity when compared to compound **90** (IC_50_ 225.8 μM). Regarding the ent-pimaradienoic acid (**91**, IC_50_ 68.7 μM) set, compound **94** (IC_50_ 3.8 μM) was the most active against trypomastigotes forms of *T. cruzi*. However, due to the lack of cytotoxicity data it is not possible to determine the selectivity index of these compounds [85].

Three quinone methide-type diterpenes (**95**–**97**) were isolated from the roots of *Salvia austriaca* Jacq. and tested for their in vitro activity against *T. b. rhodesiense* and *T. cruzi*. Cytotoxicity was determined on L6 cells [86]. The diterpene **95** was the most active and selective against *T. b. rhodesiense* trypomastigotes (IC_50_ 0.05 µM and SI = 38). However, despite exhibiting an IC_50_ value of 7.11 µM against *T. cruzi* amastigotes, its selectivity was very low (SI = 0.27). Compounds **96** and **97** also showed activity against both parasites, being more active against *T. b. rhodesiense* with IC_50_ values of 0.62 µM (SI = 5.0) and 1.67 µM (SI = 2.4), respectively. Regarding their activity against *T. cruzi*, an IC_50_ value of 7.76 µM (SI = 0.4), and 7.63 µM (SI = 0.5) was observed for compound **96** and **97**, respectively [86], but the compounds were not selective to the parasite [86].

The phytochemical study of dichloromethane extract of *Aldama discolors* (Baker) E.E.Schill. & Panero leaves led to the isolation of four structurally and biosynthetically related diterpenes. These were evaluated for in vitro activity against *T. b. rhodesiense* trypomastigotes and *T. cruzi* amastigotes [87]. Among the isolated diterpenes, compound **98** showed a moderate in vitro activity with an IC_50_ value of 15.4 µM against *T. cruzi*, and an IC_50_ 24.3 µM for *T. b. rhodesiense*. On the other hand, compound **99**, structurally similar to **98**, showed less activity against the amastigote forms of *T. cruzi* (IC_50_ 19.4 µM), and no activity against the trypomastigote forms of *T. b. rhodesiense*. The selectivity of these compounds was very low, with SI values ranging from 2 to 4 [87].

The commercially available dehydroabietylamine (**100**), an abietane-type diterpenoid isolated in large amount from *Plectranthus* genus, was used as a starting material to produce a set of amides derivatives. Among these compounds, **100**–**103** were tested against *T. cruzi* amastigotes, showing compound **103** the highest antitrypanosomal activity and selectivity (IC_50_ 0.6 μM; SI = 58). The remaining compounds, including **100**, in spite of displaying antitrypanosomal activity (IC_50_ values between 3.7 and 7.4 μM) were not very selective to the parasite, showing CC_50_ values ranging from 6.5 to 33.5 μM when tested on the human cell line (SI values between 1 and 6, L6 rat myoblasts) [88].

Leriifolione (**104**), isolated from the lipophilic extract of the roots of *Salvia leriifolia* Benth., showed high activity against *T. b. rhodesiense* (IC_50_ 1.0 μM) and *T. cruzi* (IC_50_ 4.6 μM), but an undesirable cytotoxicity on L6 cells (SI = 2.6 and 0.6, respectively) [89].

From the *n*-hexane extract of the roots of *Zhumeria majdae* Rech. F., eight abietane-type diterpenes were isolated. The antitrypanosomal activity was investigated for compounds **105**, **106**, and **107** against *T. b. rhodesiense* and *T. cruzi*. All the compounds showed a high activity against *T. b. rhodesiense*, (IC_50_ = 3.6 µM, 1.8 µM, and 0.1 µM to **105**, **106**, and **107**, respectively), being compound **106** the most selective for the parasite when compared with L6 cell line tested (**105**, SI = 1.7; **106**, SI = 21.9; **107**, SI = 15.4). None of the compounds were active against *T. cruzi* [90].

Seventeen diterpenes were isolated from the aerial parts of *Perovskia abrotanoides* Kar. These compounds were evaluated for antiprotozoal activity (*T. cruzi* amastigotes, and *T*. *b rhodesiense* trypomastigotes), and cytotoxicity was also assessed on rat skeletal myoblast L6 cell line. Most of the diterpenes were less active against *T. cruzi* than against *T. b rhodesiense* [91]. Compound **108** showed the best activity against *T. b. rhodesiense* (IC_50_ 0.5 µM, SI = 10.5). However, it was inactive against *T. cruzi*, showing a lack of selectivity (IC_50_ 58.7 µM; SI = 0.1, CC_50_ 5.2 µM). With similar activity, but less cytotoxic (CC_50_ 12.1 µM) than **108**, compound **109** displayed an IC_50_ value of 0.8 µM (SI = 14.9) against *T*. *b. rhodesiense* and an IC_50_ of 34.7 µM (SI = 0.3) against *T. cruzi*. Compounds **110**–**113** were particularly active against *T. b. rhodesiense*, displaying IC_50_ values ranging from 3.8 to 12 µM but low SI values (SI = 1.5–12.5) [91].

Bokkosin (**114**), a new cassane diterpene isolated from the Nigerian species *Calliandra portoricensis* Hassk. used in traditional medicine to treat tuberculosis, and helmintic diseases, showed a strong trypanocidal activity against the bloodstream forms of *T. b. brucei*, sensitive (IC_50_ = 1.1. μM) and resistant to pentamidine (IC_50_ = 0.5 μM). A highly favorable selectivity for the parasite strains was also observed, when compared with its cytotoxic effect on two mammalian cell lines (CC_50_ = 269 μM; SI = 246, on U937; CC_50_ = 230 μM; SI = 215, on RAW 246.7) [92].

From the *n*-hexane and ethylacetate extracts of the roots of *Acacia nilotica* L. several diterpenes were isolated. Among them, the cassane-type diterpenoid **115** was tested against the *T. b brucei* bloodstream form, exhibiting a high activity a (IC_50_ 1.4 μM). Additionally, **115** was tested for its cytotoxic effect on human HEK cells (CC_50_ = 29.5 μM; SI = 21.1) displaying a significant selectivity for the parasite [93].

### 3.4. Triterpenes

Ursolic acid (**116**) was tested for antitrypanosomal activity against *T. brucei* trypomastigotes displaying an IC_50_ value of 3.35 μM. Similar IC_50_ values were also obtained by Catteau et al**.** (IC_50_ 2.4 μM, CC_50_ > 11.1 μM). However, it is important to note that the compound showed a lack of selectivity for the trypanosoma parasite due to its cytotoxic effect against the mammalian WI38 cells used [94]. In order two find out a possible mode of action, in silico molecular modelling studies were also performed using the parasitic enzymes of the trypanosome, namely trypanothione reductase, methionyl-tRNA synthetase, and inosine-adenosine-guanosine nucleoside hydrolase [95]. Ursolic acid showed a good binding affinity for trypanothione reductase and methionyl-tRNA synthetase, which was higher than that obtain for the reference drug difluoromethylornithine. On the other hand, no inhibition was observed for inosine–adenosine–guanosine. These data may suggest that the inhibition of the two former enzymes may be responsible for the antitrypanosomal activity of compound **116** [95].

A new ursane-type triterpenoid glycoside (**117**) isolated from the dried roots of *Vangueria agrestis* (Schweinf. ex Hiern) Lantz exhibited a considerable growth suppressing effect against *T. brucei* trypomastigotes (IC_50_ 11.1 μM and IC_90_ 12.3 μM). However, no cytotoxicity studies on mammalian cell lines were performed [96].

Betulin acid (**118**), a lupane-type pentacyclic triterpene, and some semysynthetic amide derivatives were tested against *T. cruzi* trypomastigotes. Compound **118** showed a moderate activity (IC_50_ 19.5 µM and SI = 18.8), while an increasing activity was observed in derivatives **119** (IC_50_ 1.8 µM, SI = 17.3), **120** (IC_50_ 5.0 µM, SI = 10.7), and **121** (IC_50_ 5.4 µM, SI = 5.3). The mechanism of action of compound **119** in trypomastigotes was studied and seemed to be associated with the death of the parasite by necrosis, characterized by the rupture of the membrane, flagellar retraction, and appearance of atypical cytoplasmic vacuoles and dilation of the Golgi cisterns. Furthermore, the amide derivatives of compound **118** act by reduction in the invasion process, as well as the development of the intracellular parasite in host cells [97]. Sousa et al**.** corroborated those mechanistic results, showing that betulinic acid was able to inhibit all the development forms of *T. cruzi* (namely epimastigotes, trypomastigotes and amastigotes) not only by using necrotic processes but also due to modifications on the parasite mitochondrial membrane potential and the increase in reactive oxygen species [98].

Six limonoids (**122**–**127**) obtained from the roots of *Pseudocedrela kotschyi* (Schweinf.) Harms were investigated for their trypanocidal activity using bloodstream forms of *T. brucei*, showing GI_50_ values ranging from 2.5 to 14.5 μM. The most active compound was **122** (GI_50_ 2.5 μM) but showed some cytotoxicity on human HL-60 cells (GI_50_ 31.5 μM). On the other hand, limonoid **125** exhibited a similar activity (GI_50_ 3.18 μM) with no cytotoxic effect on the mammalian cell line (GI_50_ > 100 μM) [99].

From *Tabernaemontana longipes* Donn.Sm., baurenol acetate (**128**) was isolated and tested for its ability to inhibit the growth of *T. brucei* bloodstream forms, showing an IC_50_ value of 3.1 μM. Baurenol (**129**) displayed a higher activity against the same parasite (IC_50_ = 2.7 μM). Both compounds showed a low effect on cellular viability on Hep G2 cells (IC_50_ > 80 μM) [100].

Four new triterpenoids (**130**–**133**), with a rare scaffold, isolated from *Salvia hydrangea* DC. ex Benth. showed antitrypanosomal activity against *T. cruzi* amastigotes with IC_50_ values ranging from 3.5 to 19.8 μM, with no significant activity against *T. b. rhodesiense* trypomastigotes All the compounds showed a modest selectivity (SI ranging from 2.4 to 10.7; L6 cell line) [101].

Two lanostane-type triterpenoids, polycarpol (**134**) and dihydropolycarpol (**135**) were isolated from *Greenwayodendron suaveolens* (Engl. & Diels) Verdc., a plant traditional used in Congo to treat malaria. Both compounds were tested against *T. b. brucei* (IC_50_ = 8.1 μM; SI < 1.0, **134**; IC_50_ = 8.1 μM; SI = 2.4, **135**), and *T. cruzi* (IC_50_ = 1.4 μM; SI = 2.0, **134**; IC_50_ = 2.4 μM; SI = 8.1, **135**), showing good activities but very low selectivity [102].

From *Buxus sempervirens* L. leaf extract, several triterpenic-alkaloid derivatives were isolated, being the majority tested for their activity against *T. b. rhodesiense*. Cytotoxicity assays were performed on mammalian L6 cells. Compounds **136**–**142** displayed high activities with IC_50_ values < 3 μM. The highest activities and selectivities for the parasite strain tested were obtained for compounds **136** (IC_50_ = 1.5 μM; SI = 25), **138** (IC_50_ = 2.3 μM; SI = 42), **141** (IC_50_ = 2.4 μM; SI = 30), and **142** (IC_50_ = 1.3 μM; SI = 33). In spite of its promising activity against the parasite (IC_50_ = 1.1 μM), compound **146** showed a lower selectivity (SI = 12). The remaining compounds **143**–**150** displayed a significant activity with IC_50_ values ranging from 3.1 μM (compound **149**) to 9.0 μM (compound **144**), but a modest selectivity to the parasite (SI < 9) [103].

## 4. Discussion

Human African trypanosomiasis and American trypanosomiasis continue to be a major public health problem, affecting a significant proportion of the world’s population, especially in tropical countries. Currently, the drugs used to treat these diseases are scarce and far from being ideal. Therefore, the discovery and development of new drugs and treatments should be a continuous process, and all possible approaches should be explored, mainly focusing on multidisciplinary collaborations. It is also important to stress that the new drugs should be affordable and easy to administrate, improving the adherence to the therapeutic protocol, and decreasing the need for patient hospitalization.

Several approaches have been considered for the development of new drugs against these diseases. Due to the high costs and slow pace of new drug discovery, one of the main strategies is the repositioning or repurposing of drugs that were developed and used to treat other diseases. Although several advantages can be addressed, including the lower risk of failing, reduced time frame for drug development, less investment and rapid return [104], drug repositioning have also major drawbacks. These include, for example, the existence of undesirable side effects, problems concerning a different target population, poor stability in conditions of high temperature and humidity, lack of oral bioavailability, and various regulatory issues and intellectual property barriers. Therefore, the development of a new drug that ideally would be suitable for combination therapy, increasing the clinical efficacy, and decreasing side effects and the development of resistance, is a goal of utmost importance [10]. Besides combinatorial chemistry, one of the main strategies for drug discovery is through the phytochemical study of plants or other sources of natural origin. The importance of natural products for the development of drug leads or actual drugs along the last three decades has been reported in various reviews [12].

The evaluation of compounds with anti-parasitic activity is usually performed by two main approaches: the target-based and the phenotypic approaches [10,104]. The target-based methodologies (Section 2.1.1 and Section 2.2.1) focus on the specific biochemical pathway of the parasites, and consist of the identification of possible molecular targets (e.g., enzymes) that are significantly involved in the disease and the screening of molecules that possibly interfere with these targets. However, regarding trypanosomiasis, restricted success has been achieved, possibly due to a lack of translation between the activity in the molecular target, and the result on the proliferation of the parasite. In fact, the number of robust and validated molecular targets against these diseases is very limited, and in addition, for several drugs currently used in clinic, the mode of action is not yet completely understood or it comprises several targets [10,104]. By far, the most widely used methodologies to identify anti-trypanosomal drugs are based on the phenotypic methods. They consist of the screening of the compounds directly against the different forms of the parasite, and most of the time, for the bioactive compounds, there is no knowledge regarding their underlying mode of action or their molecular target. Through this screening, the effects on the parasite and the host cell viability (toxicity) can be assessed simultaneously [10].

In this review, 150 terpenic compounds obtained by isolation or derivatization from different plant species, were grouped into four classes of terpenes, namely, monoterpenes and iridoids, sesquiterpenes, diterpenes and triterpenes. The scope of this review was limited to compounds that exhibited in vitro or in vivo activity against the diverse forms of *T. brucei* and/or *T. cruzi*, displaying IC_50_ values in low micromolar range, most of them below 10 μM.

**Figure 16:**
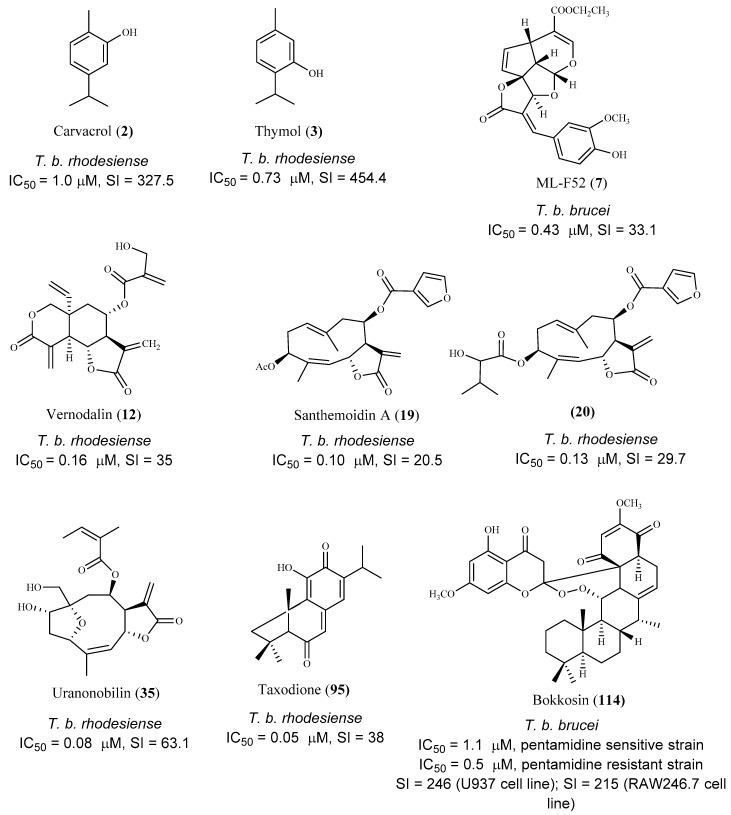
Promising hit compounds for further research into treating Human African Trypanosomiasis.

Regarding the anti-*T. brucei* activity, it can be observed that most of the selected compounds were active on the trypomastigote bloodstream form of the parasite (in vitro assays), and several possible hits can be identified. A high number of compounds exhibited very low IC_50_ values (0.05 < IC_50_ < 3.0 μM), when tested against *T. b. rhodesiense* or *T. b. brucei*, also presenting low cytotoxicity on the mammalian cells (SI values higher than 10). Some of the most promising hits are depicted in Figure 16. It is interesting to note that the majority of the bioactive compounds are sesquiterpenes and more specifically, sesquiterpenic lactones. Indeed, the α,β-unsaturated lactone function is very common in biologically active molecules, being responsible not only for their activity but also for the cytotoxicity of the compounds. The presence of this chemical function promotes the Michael-type addition to a suitable nucleophile (for example, the thiol group in proteins), and may irreversibly alkylate critical enzymes and transcription factors that control gene regulation, protein synthesis, cell metabolism, and ultimately, the cell division [105,106]. After the preliminary in vitro studies, two compounds (**3** and **7**) were further evaluated using in vivo studies on *T. brucei* mouse models achieving very good results.

**Figure 17:**
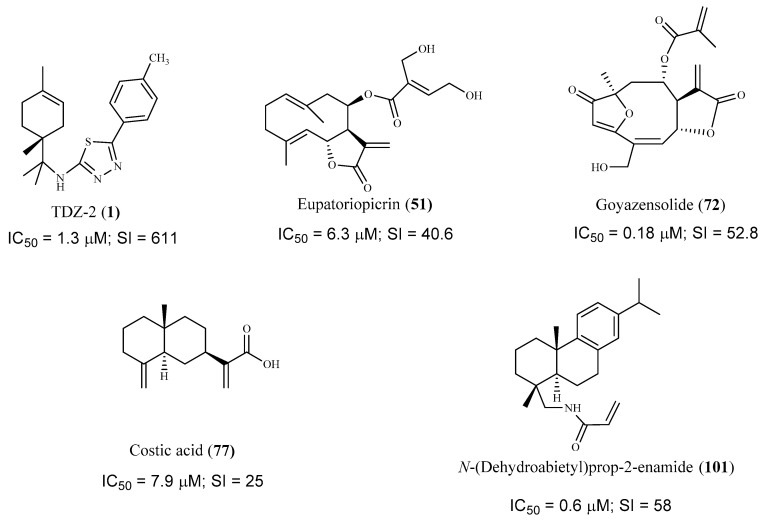
Promising hit compounds for further research into treating Human American Trypanosomiasis. All the selected compounds were tested against *T. cruzi* intracellular amastigote forms.

Concerning *T. cruzi* assays, it is curious to notice that there are much fewer research papers reporting the bioactivity of compounds against this parasite, probably because the in vitro assays using intracellular amastigotes were not so straightforward. Nevertheless, it was possible to select some promising hits that were very active against *T. cruzi* amastigotes, according to the criteria established by DNDi (Figure 17). Some of the most active compounds (**1**, **51**, and **74**) were further evaluated in vivo using a *T. cruzi* mouse model. The best result was obtained for compound **51**, and a significant reduction in parasitemia levels was observed in treated mice (1 mg/Kg/day, for 5 consecutive days), similarly to that obtained with the control group treated with the reference drug benznidazole.

Although most of the reported compounds were remarkably active against some infectious forms of the parasites, some of them also displayed some cytotoxicity on the mammalian cells tested. Furthermore, there is a notorious lack of additional studies on structure-activity relationships (SAR) and on the possible mechanisms of action. In addition, some reasons could be addressed to justify the absence of results for in vivo assays, including that the majority of compounds are isolated in very low amounts, a fact that precludes this type of assay where a large amount of compound is always needed.

## 5. Conclusions

The data presented in this review gathered the recent scientific research and experimental evidence on the most promising terpenoids derived from plants, and active against *T. brucei* and *T. cruzi* parasites. These data represent the immense efforts of various research groups all over the world, and ultimately the collected information is highly pertinent and can be used, for example, to support the selection of other plants to study, using the chemotaxonomic approach. However, all of these studies are strictly academic and no further translation to drug development has been achieved. There is still a limited collaboration of academic institutions with the pharmaceutical industry, and importantly, obtaining opportunities for research funding is, nowadays, even more challenging. All these aspects have limited or made it difficult for academic researchers to advance promising hits for further development.

In the future, natural products research must be a multidisciplinary process combining phytochemistry with innovative technological resources, in a way that will be significantly different from the past. These technologies must include high-throughput screening and in silico methodologies, as well as new extraction and dereplication procedures, new analytical tools, metabolic engineering, omics-based analysis, informatics, and big data analysis, in order to overcome the constraints of the classic natural products research.

## Figures and Tables

**Table 1:** Plant terpenoids with anti-*T. brucei* activity (2016–2021).

Compound ^a^	Plant	Parasite (Form) ^b^	IC_50_ (µM)	SI	Refs.
**Monoterpenes and Iridoids**					
carvacrol (**2**)	*Origanum onites* L.	*T. b. rhodesiense* (tryp.)	1.0	327.5	[54]
thymol (**3**)	*Origanum onites* L.	*T. b. rhodesiense* (tryp.)	0.73	454.4	[54]
terpinolene (**4**)	*Crithmum maritimum* L.	*T. brucei* (tryp.)	0.26	180.0	[55]
α-pinene (**5**)	*Crithmum maritimum* L.	*T. brucei* (tryp.)	7.4	>100	[55]
β-ocimene (**6**)	*Crithmum maritimum* L.	*T. brucei* (tryp.)	8.0	>91	[55]
ML-F52 (**7**)	*Morinda lucida* Benth.	*T. b. brucei* (tryp.)	0.43	33.1	[56]
molucidin (**8**)	*Morinda lucida* Benth.	*T. b. brucei* (tryp.)	1.27	3.7	[56]
ML-2-3 (**9**)	*Morinda lucida* Benth.	*T. b. brucei* (tryp.)	3.75	>13.3	[56]
**Sesquiterpenes**					
tagitinin C (**10**)	*Tithonia diversifolia* (Hemsl) A. Grey	*T. brucei* (tryp.)	0.012	3.0	[57]
tagitinin A (**11**)	*Tithonia diversifolia* (Hemsl) A. Grey	*T. brucei* (tryp.)	0.97	1.3	[57]
vernodalin (**12**)	*Vernonia cinerascens* Sch.Bip.	*T. b. rhodesiense* (tryp.)	0.16	35.0	[58,59]
11β, 13-dihydrovernolide (**13**)	*Vernonia cinerascens* Sch.Bip.	*T. b. rhodesiense* (tryp.)	0.5	13.0	[58,59]
11β, 13-dihydrovernodaline (**14**)	*Vernonia cinerascens* Sch.Bip.	*T. b. rhodesiense* (tryp.)	1.1	4.2	[58,59]
vernocinerascolide (**15**)	*Vernonia cinerascens* Sch.Bip.	*T. b. rhodesiense* (tryp.)	4.8	27.0	[58,59]
11β, 13-dihydrohydroxtvernolide (**16**)	*Vernonia cinerascens* Sch.Bip.	*T. b. rhodesiense* (tryp.)	5.0	4.3	[58,59]
parthenolide (**17**)	*Tarchonanthus camphoratus* L.	*T. b. rhodesiense* (tryp.)	0.39	18.6	[60]
3-oxo-1,2-dehydrocostic acid (**18**)	*Tarchonanthus camphoratus* L.	*T. b. rhodesiense* (tryp.)	2.8	6.2	[60]
santhemoidin A (**19**)	*Schkuhria pinnata* (Lam.) Kuntze ex Thell.	*T. b. rhodesiense* (tryp.)	0.10	20.5	[60]
3β-(2″-hydroxyisovaleroyloxy)-8β-(3-furoyloxy)costunolide (**20**)	*Schkuhria pinnata* (Lam.) Kuntze ex Thell.	*T. b. rhodesiense* (tryp.)	0.13	29.7	[60]
2″-dehydroeucannabinolidesemiacetal (**21**)	*Schkuhria pinnata* (Lam.) Kuntze ex Thell.	*T. b. rhodesiense* (tryp.)	0.35	11.5	[60]
3-desacetyl-3-isovaleroyleu-cannabinolide (**22**)	*Schkuhria pinnata* (Lam.) Kuntze ex Thell.	*T. b. rhodesiense* (tryp.)	0.52	13.0	[60]
3-oxo-4β,15-dihydroliqustrin-[4′,5′-dihydroxytigloyloxy] (**23**)	*Schkuhria pinnata* (Lam.) Kuntze ex Thell.	*T. b. rhodesiense* (tryp.)	0.60	19.2	[60]
schkuhrin II (**24**)	*Schkuhria pinnata* (Lam.) Kuntze ex Thell.	*T. b. rhodesiense* (tryp.)	0.82	13.4	[60]
1(10)-epoxy-3β-hydroxy-8β-[5′-hydroxy-4′-(2″- hydroxyisovaleroyloxy)tigloyloxy]costunolide (**25**)	*Schkuhria pinnata* (Lam.) Kuntze ex Thell.	*T. b. rhodesiense* (tryp.)	0.91	15.8	[60]
eucannabinolide (**26**)	*Schkuhria pinnata* (Lam.) Kuntze ex Thell.	*T. b. rhodesiense* (tryp.)	0.92	15.8	[60]
2′(3′)-*Z*-eucannabinolide (**27**)	*Schkuhria pinnata* (Lam.) Kuntze ex Thell.	*T. b. rhodesiense* (tryp.)	1.7	31.1	[60]
**28**	*Achillea fragrantissima* (Forssk.) Sch.Bip.	*T. b. brucei* (tryp.)	3.03	n.d	[61]
**29**	*Achillea fragrantissima* (Forssk.) Sch.Bip.	*T. b. brucei* (tryp.)	10.97	n.d	[61]
**30**	*Achillea fragrantissima* (Forssk.) Sch.Bip.	*T. b. brucei* (tryp.)	10.97	n.d	[61]
isofuranodiene (**34**)	*Smyrnium olusatrum* L.	*T. brucei* (tryp.)	3.0	30.0	[63]
furanonobilin (**35**)	*Anthemis nobilis* L.	*T. b. rhodesiense* (tryp.)	0.08	63.1	[64]
hydroxyisonobilin (**36**)	*Anthemis nobilis* L.	*T. b. rhodesiense* (tryp.)	0.61	8.3	[64]
8-tigloylhydroxyisonobilin (**37**)	*Anthemis nobilis* L.	*T. b. rhodesiense* (tryp.)	0.36	14.1	[64]
3-*epi*-hydroxyisonobilin (**38**)	*Anthemis nobilis* L.	*T. b. rhodesiense* (tryp.)	0.88	8.3	[64]
nobilinon A (**39**)	*Anthemis nobilis* L.	*T. b. rhodesiense* (tryp.)	0.4	3.8	[64]
**45**	Scleria striatinux De Wild.	*T. b. rhodesiense* (tryp.)	0.002	8.3	[67]
**46**	*Scleria striatinux* De Wild.	*T. b. rhodesiense* (tryp.)	0.025	3.4	[67]
vernolepin (**47**)	*Vernonia lasiopus* (O.Hoffm.) H.Rob.	*T. b. rhodesiense* (tryp.)	0.185	14.5	[59]
vernodalol (**48**)	*Vernonia lasiopus* (O.Hoffm.) H.Rob.	*T. b. rhodesiense* (tryp.)	0.26	14.4	[59]
vernomenin (**49**)	*Vernonia lasiopus* (O.Hoffm.) H.Rob.	*T. b. rhodesiense* (tryp.)	0.51	4.5	[59]
8-desacylvernodalol (**50**)	*Vernonia lasiopus* (O.Hoffm.) H.Rob.	*T. b. rhodesiense* (tryp.)	2.53	13.7	[59]
4,15-*iso*-atriplicolide methacrylate (**56**)	*Helianthus tuberosus* L.	*T. b. rhodesiense* (tryp.)	0.077	6.7	[70]
4,15-*iso*-atriplicolide isobutryrate (**57**)	*Helianthus tuberosus* L.	*T. b. rhodesiense* (tryp.)	0.26	3.38	[70]
heliantuberolide-8-O-tiglate is (**58**)	*Helianthus tuberosus* L.	*T. b. rhodesiense* (tryp.)	0.92	4.24	[70]
**78**	*Tanacetum sonbolii* Mozaff.	*T. b. rhodesiense* (tryp.)	5.1	3.9	[77]
**79**	*Tanacetum sonbolii* Mozaff.	*T. b. rhodesiense* (tryp.)	10.2	4.0	[77]
**Diterpenes**					
andrographolide *(***88***)*	*Andrographis paniculate* (Burm. F.) Wall. Ex Nees	*T. brucei* procyclic trypomastigotes	8.3	8.5	[83]
taxodione (**95**)	*Salvia austriaca* Jacq.	*T. b. rhodesiense* (tryp.)	0.05	38.0	[86]
7-(20-oxohexyl)-taxodione (**96**)	*Salvia austriaca* Jacq.	*T. b. rhodesiense* (tryp.)	0.62	5.0	[86]
taxodone (**97**)	*Salvia austriaca* Jacq.	*T. b. rhodesiense* (tryp.)	1.67	2.4	[86]
*ent*-7-oxo-pimara-8,15-diene-18-ol **(98**)	*Aldama discolors* (Baker) E.E.Schill. & Panero	*T. b. rhodesiense* (tryp.)	24.3	2.0	[87]
leriifolione (**104**)	*Salvia leriifolia* Benth.	*T. b. rhodesiense* (tryp.)	1.0	2.6	[89]
12, 16-dideoxy aegyptinone B (**105**)	*Zhumeria majdae* Rech. F	*T. b. rhodesiense* (tryp.)	3.6	1.7	[90]
11,14-dihydroxy-8, 11,13- abietatrien-7-one (**106**)	*Zhumeria majdae* Rech. F	*T. b. rhodesiense* (tryp.)	1.8	21.9	[90]
lanugon Q (**107**)	*Zhumeria majdae* Rech. F	*T. b. rhodesiense* (tryp.)	0.1	15.4	[90]
miltiodiol (**108**)	*Perovskia abrotanoides* Kar.	*T. b. rhodesiense* (tryp.)	0.5	10.5	[91]
7α-ethoxyrosmanol (**109**)	*Perovskia abrotanoides* Kar.	*T. b. rhodesiense* (tryp.) (trypomastigote)	0.8	14.9	[91]
rosmanol (**110**)	*Perovskia abrotanoides* Kar.	*T. b. rhodesiense* (tryp.)	3.8	1.5	[91]
carnosol (**111**)	*Perovskia abrotanoides* Kar.	*T. b. rhodesiense* (tryp.)	5.4	2.4	[91]
Δ^9^-dehydro-ferruginol (**112**)	*Perovskia abrotanoides* Kar.	*T. b. rhodesiense* (tryp.)	7.2	12.3	[91]
11,12-dihydroxy-20-norabieta-5(10),8,11,13-tetraen-1-one (**113**)	*Perovskia abrotanoides* Kar.	*T. b. rhodesiense* (tryp.)	12.0	12.5	[91]
bokkosin (**114**)	*Calliandra portoricensis* Hassk.	*T. b. brucei* ((ryp.)	1.1	246	[92]
8-oxacassa-13,15-dien-7-ol-17-al (**115**)	*Acacia nilotica* L.	*T. b. brucei* (tryp.)	1.4	21.1	[93]
**Triterpenes**					
ursolic acid (**116**)	*Vitellaria paradoxa* C. F. Gaertn	*T. brucei* (tryp.)	2.4	4.6	[94]
3-O-[α-L-rhamnopyranosyl-(1→2)-β-D- xylopyranosyl]pomolic acid (**117**)	*Vangueria agrestis* (Schweinf. ex Hiern) Lantz	*T. brucei* (tryp.)	11.1	n.d.	[96]
kotschyienone A (**122**)	*Pseudocedrela kotschyi* (Schweinf.) Harms	*T. brucei* (tryp.)	2.5	12.6	[99]
7-deacetyl-7-oxogedunin (**125**)	*Pseudocedrela kotschyi* (Schweinf.) Harms	*T. brucei* (tryp.)	3.18	>31.4	[99]
baurenol acetate (**128**)	*Tabernaemontana longipes* Donn.Sm.	T. brucei (tryp.)	3.1	>25.8	[100]
baurenol (**129**)	Tabernaemontana longipes Donn.Sm.	T. brucei (tryp.)	2.7	>29.6	[100]
polycarpol (**134**)	Greenwayodendron suaveolens (Engl. & Diels) Verdc.	*T. b. brucei* (tryp.)	8.1	1.0	[102]
dihydropolycarpol (**135**)	*Greenwayodendron suaveolens* (Engl. & Diels) Verdc.	*T. b. brucei* (tryp.)	8.1	2.4	[102]
cyclovirobuxeine-B (**136**)	*Buxus sempervirens* L.	*T. b. rhodesiense* (tryp.)	1.5	25.0	[103]
cyclomicrophylline-A (**138**)	*Buxus sempervirens* L.	*T. b. rhodesiense* (tryp.)	2.3	42.0	[103]
N-benzoyl-O-acetyl-cycloxo-buxoline-F (**141**)	*Buxus sempervirens* L.	*T. b. rhodesiense* (tryp.)	2.4	30.0	[103]
N20-acetylbuxadine- G (**142**)	*Buxus sempervirens* L.	*T. b. rhodesiense* (tryp.)	1.3	33.0	[103]
O-benzoyl-cycloprotobuxoline-D (**146**)	*Buxus sempervirens* L.	*T. b. rhodesiense* (tryp.)	1.1	12.0	[103]

^a^ Some names are not indicated in the corresponding papers; ^b^ Tryp: trypomastigotes bloodstream forms: n.d.: not defined/not determined.

**Table 2:** Plant terpenoids with anti-*T. cruzi* activity (2016–2021).

Compound ^a^	Plant	Parasite Form	IC_50_ (µM)	SI	Refs.
**Monoterpenes and Iridoids**					
N-{1-methyl-1-[(1R)-4-methylcyclohex-3-en-1- yl]ethyl}-5-(4-methylphenyl)-1,3,4-thiadiazol-2-amine (**1**)	n.d	Amastigote	1.3	611.2	[53]
**Sesquiterpenes**					
mikanolide (**31**)	*Mikania variifolia* Hieron. and *Mikania micrantha* Kunth	Epimastigote Trypomastigote Amastigote	2.41 7.24 15.5	31.9 10.6 4.3	[62]
deoxymikanolide (**32**)	*Mikania variifolia* Hieron. and *Mikania micrantha* Kunth	Epimastigote Trypomastigote Amastigote	0.29 5.43 22.8	992.5 54.0 12.5	[62]
dihydromikanolide (**33**)	*Mikania micrantha* Kunth	Epimastigote Trypomastigote Amastigote	8.55 1.03 29.1	5.2 49.0 1.5	[62]
nobilinon A (**39**)	*Anthemis nobilis* L.	Intracellular amastigote	2.8	0.5	[64]
nobilinon B (**40**)	*Anthemis nobilis* L.	Intracellular amastigote	4.2	6.1	[64]
11,13-dihydroxy-calaxin (**41**)	*Calea pinnatifida* (R. Br.) Less.	Amastigote	8.30	1.88	[65]
enhydrin (**42**)	*Smallanthus sonchifolius* (Poepp.) H. Rob.	Epimastigote	0.78	n.d.	[66]
uvedalin (**43**)	*Smallanthus sonchifolius* (Poepp.) H. Rob.	Epimastigote	0.79	n.d.	[66]
polymatin (**44**)	*Smallanthus sonchifolius* (Poepp.) H. Rob.	Epimastigote	1.38	n.d.	[66]
**45**	*Scleria striatinux* De Wild.	Amastigote	0.025	0.74	[67]
**46**	*Scleria striatinux* De Wild.	Amastigote	0.085	1.0	[67]
eupatoriopicrin (**51**)	Astearaceae species	Trypomastigote bloodstream form Intracellular amastigote	19.9 6.3	12.9 40.6	[68]
estafietin (**52**)	Astearaceae species	Trypomastigote bloodstream form	117	6.8	[68]
eupahakonenin B (**53**)	Astearaceae species	Trypomastigote bloodstream form Intracellular amastigote	33.0 89.3	10.4 3.8	[68]
minimolide (**54**)	Astearaceae species	Trypomastigote bloodstream form Intracellular amastigote	21.0 25.1	12.8 10.7	[68]
tagitinin C (**55**)	*Tithonia diversifolia* (Hemsl.) A. Gray	Epimastigote	1.15	5.69	
4,15-iso-atriplicolide methacrylate (**56**)	*Helianthus tuberosus* L.	Trypomastigote	1.6	0.32	[73]
4,15-iso-atriplicolide isobutryrate (**57**)	*Helianthus tuberosus* L.	Trypomastigote	3.1	0.28	[73]
heliantuberolide-8-O-tiglate (**58**)	*Helianthus tuberosus* L.	Trypomastigote	5.7	0.68	[73]
(2-methoxy-2,5-epoxy-8-metha- cryloxygermacra-3Z,11(13)-dien-6,12-olide (**59**)	*Vernonanthura nebularum* (Cabrera) H. Rob.	Epimastigote	1.5	> 14	[71]
(2-ethoxy-2, 5-epoxy-8-angeloxygermacra-3Z,11(13)-dien-6,12-olide (**60**)	*Vernonanthura nebularum* (Cabrera) H. Rob.	Epimastigote	2.1	> 14	[71]
8a-methacryloxyhirsutinolide 13-O-acetate (**61**)	*Vernonanthura nebularum* (Cabrera) H. Rob.	Epimastigote	2.0	> 14	[71]
**62**	*Vernonanthura nebularum* (Cabrera) H. Rob.	Epimastigote	3.7	14.3	[71]
**66**	*Vernonanthura nebularum* (Cabrera) H. Rob.	Epimastigote	10.7	9.0	[71]
**67**	*Vernonanthura nebularum* (Cabrera) H. Rob.	Epimastigote	8.1	13.9	[71]
**68**	*Centratherum puctatum* ssp. Punctatum Cass.	Epimastigote	6.8	1.6	[71]
**69**	*Elephantopus mollis* Kunth	Epimastigote	4.7	11.5	[71]
eucannabinolide (**70**)	*Urolepis hecatantha* (DC.) R.King & H.Rob.	Epimastigote	10	1.5	[72]
santhemoidin C (**71**)	*Urolepis hecatantha* (DC.) R.King & H.Rob.	Epimastigote	18	0.83	[72]
goyazensolide (**72**)	*Lychnophora passerina* (Mart ex DC) Gardn.	Intracellular amastigote	0.181/24 h 0.020/48 h	52.82/24h 915.0/48h	[73]
hedyosulide (**73**)	*Hedyosmum brasiliense* Mart. ex Miq.	Trypomastigote Intracellular amastigote	28.1 21.6	>7 >9	[74]
8-epi-xanthatin-1β,5β-epoxide (**75**)	*Inula viscosa* (L.) Greuter	Epimastigote	4.99	3.67	[75]
inuloxin A (**76**)	*Inula viscosa* (L.) Greuter	Epimastigote	15.52	3.38	[75]
costic acid (**77**)	*Nectandra barbellata* Coe-Teix.	Intracellular amastigote	7.9	>25	[76]
polygodial (**80**)	*Polygonum hydropiper* L.	Epimastigote Trypomastigote Amastigote	51.0 68.2 34.4	n.d	[79]
Polygodial derivative (**81**)	n.d	Epimastigote Trypomastigote Amastigote	13.0 8.4 9.9	n.d	[79]
Polygodial derivative (**82**)	n.d	Epimastigote Trypomastigote Amastigote	12.3 6.4 6.7	n.d	[79]
Polygodial derivative (**83**)	n.d	Epimastigote Trypomastigote Amastigote	7.2 6.9 8.3	n.d	[79]
epi-polygodial (**84**)	*Drimys brasiliensis* Miers	Trypomastigote	5.01	>40	[80]
(-)-T-cadinol (**85**)	*Casearia sylvestris* Sw.	Trypomastigote Amastigote	18.2 15.8	>15	[81]
**Diterpenes**					
ent-15β-senecioyl-oxy-kaur-16-en-19-oic acid (**89**)	*Baccharis retusa* DC.	Trypomastigote	3.8	50.0	[84]
taxodione (**95**)	*Salvia austriaca* Jacq.	Amastigote	7.11	0.27	[86]
7-(20-oxohexyl)-taxodione (**96**)	*Salvia austriaca* Jacq.	Amastigote	7.76	0.4	[86]
Taxodone (**97**)	*Salvia austriaca* Jacq.	Amastigote	7.63	0.5	[86]
**ent**-7-oxo-pimara-8,15-diene-18-ol (**98**)	*Aldama discolors* (Baker) E.E.Schill. & Panero	Amastigote	15.4	3.0	[87]
ent-2S,4S-2-19-epoxy-pimara-8(3),15-diene-7β-ol (**98**)	*Aldama discolors* (Baker) E.E.Schill. & Panero	Amastigote	19.4	4.0	[87]
dehydroabietylamine derivative(**103**)	n.d	Amastigote	0.6	58.0	[88]
Leriifolione (**104**)	*Salvia leriifolia* Benth.	Amastigote	2.6	0.6	[89]
**Triterpenes**					
Betulinic acid (**118**)	n.d	trypomastigote	19.5	18.8	[97]
Betulinic acid derivative (**119**)	n.d	trypomastigote	1.8	17.3	[97]
Betulinic acid derivative (**120**)	n.d	trypomastigote	5.0	10.7	[97]
Betulinic acid derivative (**121**)	n.d	trypomastigote	5.4	5.3	[97]
Perovskone C (**130**)	*Salvia hydrangea* DC. ex Benth.	Amastigote	3.5	10.7	[101]
perovskone D (**131**)	*Salvia hydrangea* DC. ex Benth.	Amastigote	3.8	3.6	[101]
perovskone E (**132**)	*Salvia hydrangea* DC. ex Benth.	Amastigote	11.5	6.3	[101]
perovskone F (**133**)	*Salvia hydrangea* DC. ex Benth.	Amastigote	19.8	2.4	[101]
*polycarpol* (**134**)	*Greenwayodendron suaveolens (Engl. & Diels) Verdc.*	trypomastigote	1.4	2.0	[102]
dihydropolycarpol (**135**)	*Greenwayodendron suaveolens* (Engl. & Diels) Verdc.	trypomastigote	2.4	8.1	[102]

^a^ Some names are not indicated in the corresponding papers; n.d.: not defined/not determined.
